# Strategies to overcome myeloid cell induced immune suppression in the tumor microenvironment

**DOI:** 10.3389/fonc.2023.1116016

**Published:** 2023-04-11

**Authors:** Jennifer Cao, Lyndah Chow, Steven Dow

**Affiliations:** ^1^ Flint Animal Cancer Center, College of Veterinary Medicine and Biomedical Sciences, Colorado State University, Fort Collins, CO, United States; ^2^ Department of Microbiology, Immunology, and Pathology, College of Veterinary Medicine and Biomedical Sciences, Colorado State University, Fort Collins, CO, United States; ^3^ Department of Clinical Sciences, College of Veterinary Medicine and Biomedical Sciences, Colorado State University, Fort Collins, CO, United States

**Keywords:** cancer, immune suppresion, tumor associate macrophages (TAM), myeloid derived suppressor cells (MDSC), tumor associated neutrophils (TAN), cancer immune therapy, tumor microenvironment, dendritc cells

## Abstract

Cancer progression and metastasis due to tumor immune evasion and drug resistance is strongly associated with immune suppressive cellular responses, particularly in the case of metastatic tumors. The myeloid cell component plays a key role within the tumor microenvironment (TME) and disrupts both adaptive and innate immune cell responses leading to loss of tumor control. Therefore, strategies to eliminate or modulate the myeloid cell compartment of the TME are increasingly attractive to non-specifically increase anti-tumoral immunity and enhance existing immunotherapies. This review covers current strategies targeting myeloid suppressor cells in the TME to enhance anti-tumoral immunity, including strategies that target chemokine receptors to deplete selected immune suppressive myeloid cells and relieve the inhibition imposed on the effector arms of adaptive immunity. Remodeling the TME can in turn improve the activity of other immunotherapies such as checkpoint blockade and adoptive T cell therapies in immunologically “cold” tumors. When possible, in this review, we have provided evidence and outcomes from recent or current clinical trials evaluating the effectiveness of the specific strategies used to target myeloid cells in the TME. The review seeks to provide a broad overview of how myeloid cell targeting can become a key foundational approach to an overall strategy for improving tumor responses to immunotherapy.

## 1 Introduction

Local immune suppression and dysregulation are common features of cancer and are closely associated with tumor metastasis and resistance to therapy. The interaction between cancer and the host immune system is a key factor in determining tumor control or progression (1–3). Tumor infiltrating leukocytes, particularly monocytes, myeloid derived suppressor cells (MDSCs) and neutrophils create a tumor microenvironment (TME) that is inhospitable to effector cells such as CD4 and CD8 T cells and NK cells (4–8). Myeloid lineage cells such as dendritic cells (DCs), tumor associated macrophages (TAMs) and MDSCs can serve a dichotomous role within the TME, though in general they are largely immune suppressive (9–12). These myeloid cells can promote tumor growth by exerting immune suppressive pressure, including secreted cytokines and growth factors promoting angiogenesis, direct cellular signaling or recruitment of Tregs and other immune suppressive cells such as TAMs, MDSCs, tumor associated neutrophils (TANs) and DCs (13). Myeloid cells in the TME can also assume a tumoricidal phenotype, as is the case with activated M1 macrophages producing free radicals and cytokines that stimulate the activation of effector T cells (14), or antigen presenting DCs that promote the expansion and activation of effector CD4 and CD8 T cells.

The net outcome of the dynamic interplay in the TME is determined in part by secreted factors and cell signaling from tumor and stromal cells and by the resident immune cells within the TME, which perpetuate either a suppressive or stimulatory immune landscape (1, 4, 10, 12). Targeting of myeloid immune suppressor cells to reduce or eliminate their immune suppressive impacts on adaptive immunity can turn the tide between cancer and the host’s immunity, thereby increasing tumor control and improving the efficacy of other treatments. In this review we summarize past and current strategies including relevant clinical trials that target myeloid cells in the TME as cancer immunotherapy strategies. Although this is not intended to be a fully comprehensive review of all strategies and trials, the goal is to emphasize that the myeloid cell component of the TME presents many opportunities for development of new immune based therapeutics.

## 2 Immune suppressive myeloid cells, origins, and key functions

### 2.1 Origin and differentiation of immune suppressor cells

Immune suppressor cells in the tumor microenvironment can be characterized by their cell type of origin. Myeloid derived suppressor cells (MDSC) are comprised of both neutrophil derived MDSC (PMN-MDSC) and monocyte derived MDSC (M-MDSC) with potent immune suppressive activity (15). Tumor associated macrophages (TAM) are derived from inflammatory monocytes recruited from the bloodstream in response to chemokines produced by tumor cells and the tumor stroma, including also myeloid cells themselves, and can be clearly distinguished phenotypically and functionally from MDSC (16) (17). The distinction between tumor associated neutrophils (TAN) and PMN-MDSC is somewhat more complicated, in that they share many phenotypic characteristics (18). Tolerogenic DCs are dendritic cells exposed to polarizing cytokines and surface molecules secreted by tumor cells and stromal cells within the tumor microenvironment (9) This population of immune suppressive DC suppress effector T cell responses, thereby contributing to an overall immune suppressed and hostile environment for infiltrating T cells.

**Figure 1:**
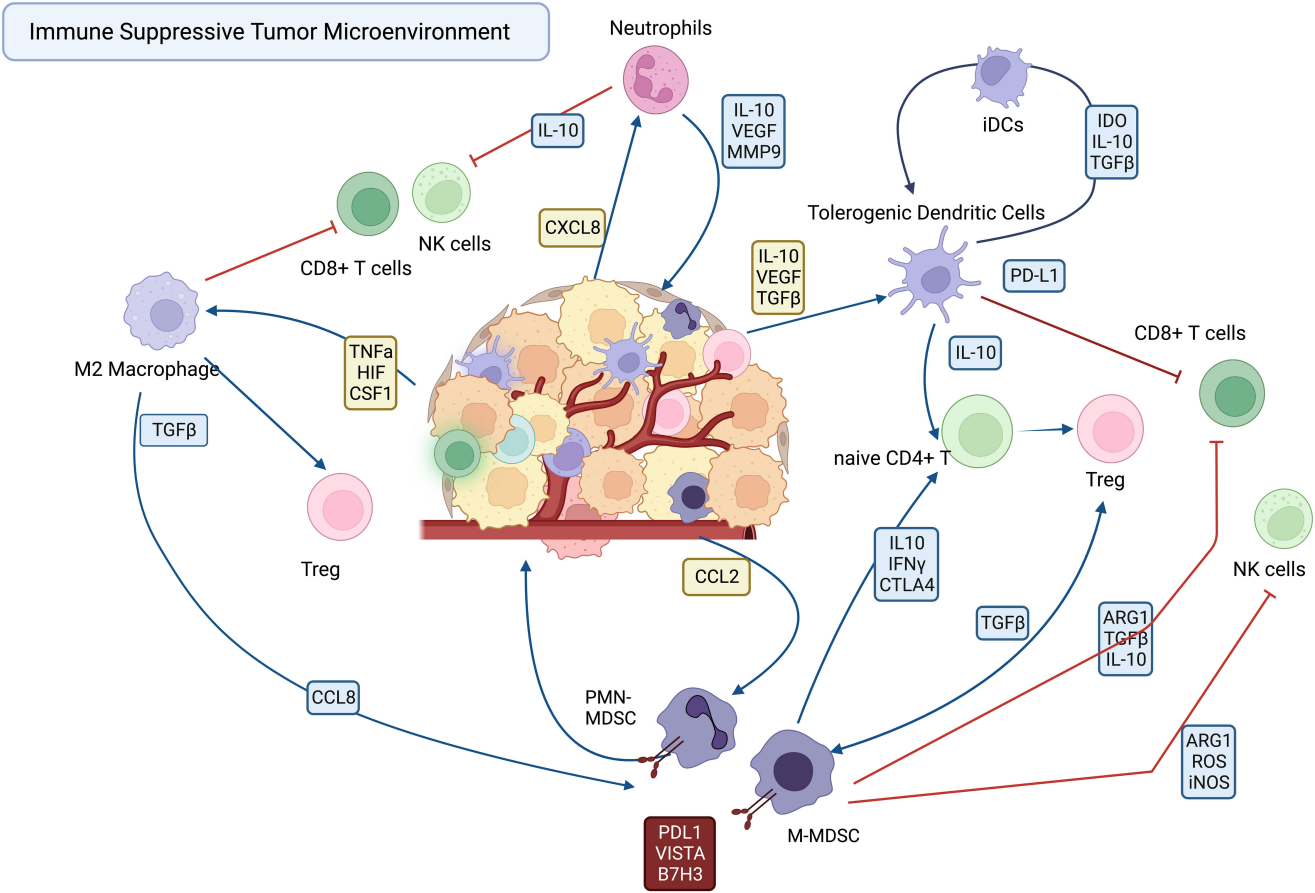
Cellular interactions in the immune suppressive tumor microenvironment.

The immune suppressive TAMs, DCs, TANs and MDSCs are recruited to the tumor by a variety of cellular and soluble factors within the tumor milieu, where they suppress effector functions of T cells and NK cells (**Figure 1**). The various mechanisms employed by immune suppressive myeloid cells become potential targets for new immunotherapies designed to reprogram the TME. Among the mechanisms employed by TAMs and MDSCs to suppress effector T cells and NK cells include upregulated expression of immune suppressive checkpoint molecules such as PD-L1 (19, 20), VISTA (21, 22), and B7-H3 ) (21, 23–25). Other mechanisms include secretion of immune suppressive cytokines such as IL10 and TGFβ, and VEGF (6, 26).

### 2.2 Function of TAMs in the tumor microenvironment

Immunologically “cold” tumors evade immune surveillance through a variety of mechanisms. Down regulation of tumor associated antigens (TAA) (27, 28), maintaining inflammation leading to immune exhaustion (6, 26) and increasing angiogenesis to tumor sites (29, 30) are all methods used by tumors to persist and metastasize while evading detection by the immune system.

There is strong clinical evidence linking TAMs to cancer immune suppression. For example, the density of TAMs infiltrating tumors is strongly correlated with poor overall survival in many breast, ovarian, bladder, gastric, thyroid and colorectal cancers (31). TAMs have a relatively short half-life and are therefore must be replaced continuously by inflammatory monocytes recruited from the bloodstream primarily in response to the chemokine CCL2, produced by tumor cells, tumor fibroblasts, and by myeloid cells (32). This dependence of TAMs on continuous monocyte replacement opens a window of opportunity for therapeutic intervention and depletion of TAMS.

Once within the tumor, the differentiation of monocytes to TAMs is guided by either pro-inflammatory or anti-inflammatory factors produced within the TME. The overwhelming majority of TAMs in most tumors exist in a state that most closely resembles that of what has been defined experimentally as M2 polarization, which results in a macrophage that is generally immune suppressive and tumor growth and metastasis promoting (3). The M2 polarization state of TAMS is driven by a a diverse array of cytokines (eg, IL-10, TGF-b), chemokines (CXCL4, CCL5), growth factors (VEGF, M-CSF) and by local tumor hypoxia (33). Tumor cells can also directly contribute metabolically to M2 polarization by secretion of lactic acid and hypoxia-inducible factor (HIF1α) (34). Tumor cells also co-opt TAM signaling to promote tumor growth locally, and to become more invasive for generating metastases. For example, tumor secretion of TNF-α induces the chemokines CCL2 and CCL8 by TAMs which recruits additional CCR2+ monocytes to the TME (35). In another example, CCL8 produced by TAMs also upregulate tumor cell secretion of colony stimulating factor 1 (CSF-1) which is crucial to macrophage and DC survival and differentiation through signaling *via* CSF-1R (36, 37). One of the most important consequences of the accumulation of TAMs is the impact on T cell effector functions. For example, TGF-β signaling drives CD4 T cell differentiation towards immune suppressive Th2 and Treg phenotypes (38). TGF-β signaling also suppresses the effector functions of CD8 T cells and NK cells and decreases migration of DCs into the tumor tissues (9). Within the TME, T cells responding to TAM secreted factors exhibit upregulated expression of immune suppressive immune checkpoint molecules such as PD-1, CTLA-4, Lag3, and TIM3 (39). The expression of the ligand for PD-1 (PDL-1) is often higher on TAMs than it is on tumor cells, and PDL-1 signaling to TAM directly can reduce their ability to phagocytose tumor cells (40).

Metabolically, TAMs can reprogram the TME by producing enzymes that directly alter T cell signaling, or deplete necessary amino acids needed for T cell survival and proliferation. For example, TAM production of arginase 1 (Arg-1) leads to the depletion of L-arginine in turn leading to dysfunction of tumor infiltrating lymphocytes by TCR ζ chain downregulation (1, 12, 41). In another example, TAM (and tumor cells) can overproduce the enzyme indoleamine dehydrogenase (IDO), which depletes the TME of tryptophan, a necessary amino acid for T cell survival (42).

### 2.3 Function of immune suppressive DCs in the tumor microenvironment

Like TAMs, DCs in the TME exist primarily in an immune suppressive state and by inactivating effector T cells can promote more rapid tumor growth and metastasis (9). Immature DCs that reside in the TME recognize tumor cells and the products of tumor cell necrosis through damage-associated molecular patterns (DAMPs) which induce DC phagocytosis and processing of tumor antigens. This process matures DCs to serve their primary role as antigen presenting cells, and stimulates migration to lymph nodes, and ultimately leading to activation or inactivation of both CD4 and CD8 effector T cells (43). Classically differentiated DCs secrete proinflammatory cytokines such as IL-12 to activate IFNγ producing T cells and NK cells, which drives differentiation of Th1 T cells and activated CD8+ cytotoxic T cells (44). However, in the TME most DCs exist in an immature state and become toleragenic DCs (tDCs) following sustained exposure to cytokines such as VEGF, IL10 and TGFβ (9, 43). tDCs induce T cell anergy through checkpoint molecule signaling, including signaling *via* CD28 and PDL-1 to their cognate receptors CTLA-4 and PD-1 on T cells (45). tDCs also promote the generation of regulatory T cells (Tregs) from naïve CD4+ T cells by secretion of IL10 and TGF-β (43).

### 2.4 MDSCs in the tumor microenvironment

MDSCs are derived from immature monocytes and neutrophils, mobilized from the bone marrow in response to cytokines associated with chronic inflammation, including IL-3, GM-CSF, and G-CSF (46). The two primary populations of MDSC are defined as monocytic MDSC (M-MDSC) and neutrophilic MDSC (PMN-MDSC), which have both distinct and overlapping molecular and functional characteristics. For example, they exhibit distinct gene expression profiles, and unique immunologic functions such as production of arginase (PMN-MDSC) or reactive nitrogen or oxygen intermediates (M-MDSC) (47). Following their mobilization from the bone marrow and entry into the bloodstream, MDSC are recruited into tumor tissues in part by following chemokine gradients such as CCL2 and CXCL8, and other cytokines secreted by tumor cells and immune cells within the TME (48). Once in the TME, MDSC can be induced to undergo further differentiation to become more immune suppressive, by factors such as TGF-b produced by Tregs. MDSCs can also accumulate in secondary lymphoid tissues including the spleen and lymph nodes where they contribute to systemic immune suppression and further promote tumor progression (38, 49).

The expansion and differentiation of Tregs within the TME, is promoted in part by MDSC expression of tumor derived peptides on MHCI and MHCII molecules (50). As another mechanism of MDSC polarization, histamine released by mast cells binds to histamine receptor 1 on MDSCs inducing secretion of Arg-1 and nitric oxide synthase (iNOS) which inhibits T cell proliferation (51). Recent studies have correlated the abundance of MDSCs with poor prognosis and poor response to immune checking inhibitor (ICI) therapy in patients with various cancer types including breast, colorectal, lung and prostate cancers (52–54).

M-MDSC were originally defined in tumor-bearing mice as immature bone marrow derived cells that suppressed multiple T cell functions (55). The population of M-MDSC overall is considered to be more immune suppressive than PMN-MDSCs despite making up only 10-20% of the total MDSC population (56). Mechanisms of M-MDSC-mediated immune suppression include production of suppressive cytokines IL10 and TGF-β (4); They also promote T cell apoptosis by TCR-ζ chain downregulation through secretion of iNOS, arginase, and and reactive oxygen species (ROS) in mouse models. Secretion of iNOS also inhibits NK cells, thereby reducing antibody-dependent cell-mediated cytotoxicity (ADCC) (57). In a clinical setting, patients with non-small cell lung carcinoma (NSCLC), circulating tyrosine kinase receptor TIE2^hi^ expressing M-MDSCs were found to suppress antigen-specificT cell responses and their presence was linked to poor patient outcomes (58). In contrast, patients with NSCLC treated with anti-PD-1 checkpoint blockade that had lower frequencies of both M-MDSCs and PMN-MDSCs had longer overall progression free survival (59).

PMN-MDSCs comprise the majority of MDSC populations (60). These PMN-MDSC are metabolically distinctive from mature neutrophils and promote early tumor dissemination and establishment of the pre-metastatic niche in the lungs and other sites (61). They also migrate more effectively and exert significantly greater immune suppressive activity compared to normal neutrophils. Mechanisms by which PMN-MDSCs inhibit T cell function include reactive nitrogen intermediates in mice and dogs, and ROS in humans (60, 62). Patients with primary and metastatic lung cancers exhibited high numbers of tumor infiltrating PMN-MDSCs, which was associated with suppressed NK cell activation and cytolytic activity, thought to be mediated by both cell-cell contact with PMN-MDSC and production of soluble factors within the TME (54).

### 2.5 Immune modulative TANs in the tumor microenvironment

Immune suppressive TANs and PMN-MDSCs are recruited to the TME primarily by the chemokine CXCL8, which signals *via* the chemokine receptors CXCL1 and CXCL2 (63). Given their common origins in the bone marrow and their shared need for growth factors and cytokines such as G-CSF, IL-6, and IL-17, it is somewhat difficult to definitely distinguish TANs from PMN-MDSCs (47, 63). Within tumor tissues, TANs are classified as either N1 or N2 populations, analogous to M1 and M2 macrophages (64). Populations of N1 TANs exhibit antitumor activity, whereas N2 TANs inhibit T cell proliferation and promote tumor growth (65). TGF-β secreted by tumor cells is one mechanism that polarizes TANs to the N2 phenotype (64, 66, 67). Within the TME, N2 TANs promote angiogenesis and play a role in establishing the pre-metastatic niche through secretion of VEGF and by expression of metalloproteinase-9 (MMP-9) which decreases the bioavailability of anti-angiogenic molecules (68, 69). High circulating numbers of N2 TANs have been associated with increased tumor metastatic progression, and genetic instability in tumors including melanoma and bronchioloalveolar carcinoma (70–72). Depletion of N2 TANs in animal models leads to increased numbers of effector CD8 T cells (73)’ and promotes their infiltration into the tumor (63). Secretion of TGF-β and IL-10 by N2 TANs stimulates MDSC expansion, further augmenting the immune suppressive nature of the TME. Importantly, the mechanisms by which N2 TANs suppress tumor immunity may in many cases be distinct and different in mice versus humans (74).

In addition to suppressing T cells, both PMN-MDSC and N2 TANs produce neutrophil extracellular traps (NETs). These complex webs, comprised of extruded DNA molecules studded with chromatin and other nuclear proteins, can promote tumor metastasis by trapping migrating tumor cells within tumor blood vessels, and then facilitating the survival of these early metastatic tumor cells (75, 76). The NETS secreted by TANs and PMN-MDSC also interrupt the cytotoxic activities of CD8+ T cells and NK cells (77).

## 3 Therapeutic targeting of immune suppressive macrophages and MDSC

### 3.1 Direct depletion of myeloid cells (TAM, MDSC) in the TME

Depletion of immune suppressive myeloid cell populations within the TME is one method to overcome the immune suppressive pressure they exert, particularly given that these cells, especially TAMs, can be quite numerous in the TME, in some cases comprising over 50% of the entire tumor cell population (78). Below we provide examples of the multiple strategies designed to deplete TME populations of immune suppressive TAMs.

#### 3.1.1 Colony-stimulating factor 1 receptor blockade

One approach that has been extensively investigated is TAM depletion *via* blocking signaling by the essential macrophage growth factor receptor colony-stimulating factor 1 receptor (CSF-1R). CSF-1R is expressed by TAMs and binds to the primary ligands CSF-1 and IL-34 (79). CSF-1R signaling is crucial to macrophage differentiation and survival (79, 80). The density of CSF-1R+ TAMs in tumors correlates with poor outcomes in many tumor types, including colon adenocarcinoma, pancreatic cancer, classical Hodgkin lymphoma, leiomyosarcoma, hepatocellular carcinoma and breast cancer (81–86). CSF-1R is also expressed by other immune cells within the TME such as DC, MDSCs and neutrophils, and blocking CSF-1R signaling may therefore also deplete these cells in addition to TAMs (87). Disruption of CSF-1R signaling has been achieved by use of small molecules and monoclonal antibodies (87, 88). Multiple clinical trials are ongoing to evaluate the effects of CSF-1/CSF-1R blockade on TAM populations and tumor control in many both solid tumors and hematologic cancers (**Table 1**). To date clinical trials for CSF-1/CSF-1R blockade have been completed in non-pancreatic neuroendocrine tumors (89), Hodgkin lymphoma (90), ovarian cancer (NCT03166891, NCT03901118), non-Hodgkin lymphoma (NCT03974243) and hepatocellular carcinoma (NCT03245190). The accumulated results from these trials indicates safety and tolerability of the CSF-1R inhibitors, but limited efficacy, suggesting either refined dosage or timing of CSF-1/CSF-1R blockade, or the need to employ with other combination therapies (91).

**Table 1:** US clinical trials in cancer using Colony-stimulating factor 1 receptor (CSF-1R) blockade as intervention.

CSF-1/CSF-1R blockade	NCT Number	Title	Status	Study Results	Conditions	Interventions	Phases	Enrollment
1	NCT03158103	A Study of MEK162 (Binimetinib) in Combination With Pexidartinib in Patients With Advanced Gastrointestinal Stromal Tumor (GIST)	Completed	No Results Available	Gastrointestinal Stromal Tumor (GIST)	MEK162| Pexidartinib	Phase 1	3
2	NCT02390752	Phase I Trial of Turalio(R) (Pexidartinib, PLX3397) in Children and Young Adults With Refractory Leukemias and Refractory Solid Tumors Including Neurofibromatosis Type 1 (NF1) Associated Plexiform Neurofibromas (PN)	Recruiting	No Results Available	Neurofibroma, Plexiform|Precursor Cell Lymphoblastic Leukemia-Lymphoma|Leukemia, Promyelocytic, Acute|Sarcoma	TURALIO	Phase 1	54
3	NCT04635111	A Long-term Study Evaluating Hepatotoxicity Associated With TURALIO (Pexidartinib) Treatment	Recruiting	No Results Available	Hepatotoxicity|Tenosynovial Giant Cell Tumor	TURALIO		30
4	NCT02371369	Phase 3 Study of Pexidartinib for Pigmented Villonodular Synovitis (PVNS) or Giant Cell Tumor of the Tendon Sheath (GCT-TS)	Completed	CR 24.2%, PR 29.7%	Pigmented Villonodular Synovitis|Giant Cell Tumors of the Tendon Sheath|Tenosynovial Giant Cell Tumor	Pexidartinib| Placebo	Phase 3	120
5	NCT04526704	Study to Evaluate Discontinuation and Re-Treatment in Participants With Tenosynovial Giant Cell Tumor (TGCT) Previously Treated With Pexidartinib	Active, not recruiting	No Results Available	Tenosynovial Giant Cell Tumor	Pexidartinib	Phase 4	32
6	NCT02401815	CGT9486 (Formerly Known as PLX9486) as a Single Agent and in Combination With PLX3397 (Pexidartinib) or Sunitinib in Participants With Advanced Solid Tumors	Completed	No Results Available	Gastrointestinal Stromal Tumors	PLX9486| Pexidartinib| Sunitinib	Phase 1|Phase 2	51
7	NCT01349036	A Phase 2 Study of PLX3397 in Patients With Recurrent Glioblastoma	Terminated	Surgical Cohort 1, Overall survival 76.9%. SD 23.1%, PD 76.9%. Non-Surgical Cohort 2 Overall survival 95.5%	Recurrent Glioblastoma	PLX3397	Phase 2	38
8	NCT02452424	A Combination Clinical Study of PLX3397 and Pembrolizumab To Treat Advanced Melanoma and Other Solid Tumors	Terminated	no CR in any dose escalation. PR up to 15.4% in Melanoma	Melanoma|Non-small Cell Lung Cancer|Squamous Cell Carcinoma of the Head and Neck|Gastrointestinal Stromal Tumor (GIST)|Ovarian Cancer	PLX3397|Biological: Pembrolizumab	Phase 1|Phase 2	78
9	NCT01790503	A Phase 1b/2 Study of PLX3397 + Radiation Therapy + Temozolomide in Patients With Newly Diagnosed Glioblastoma	Completed	CR + PR up to 18.2%, SD up to 54.5%	Patients With Newly Diagnosed Glioblastoma	PLX3397|Radiation: Radiation Therapy| Temozolomide	Phase 1|Phase 2	65
10	NCT01525602	Safety Study of PLX3397 and Paclitaxel in Patients With Advanced Solid Tumors	Completed	Clinical benefit rate (CR, PR, or stable disease) 33~ 67%	Solid Tumors	PLX3397| Paclitaxel	Phase 1	74
11	NCT05271292	Chiauranib for Advanced Solid Malignant Tumors and Relapsed/Refractory SCLC.	Recruiting	No Results Available	Small-cell Lung Cancer|Advanced Solid Malignant Tumor	Chiauranib	Phase 1|Phase 2	36
12	NCT01316822	A Study of ARRY-382 in Patients With Selected Advanced or Metastatic Cancers	Completed	No Results Available	Metastatic Cancer	ARRY-382, cFMS inhibitor; oral	Phase 1	26
13	NCT02880371	A Study of ARRY-382 in Combination With Pembrolizumab for the Treatment of Patients With Advanced Solid Tumors	Terminated	Phase 1b, 10.5% had confirmed PR, in phase 2, 3.7%with PDA had a PR lasting 2.4 months.	Advanced Solid Tumors	ARRY-382|Pembrolizumab	Phase 1|Phase 2	82
14	NCT01804530	Phase 1 Study of PLX7486 as Single Agent in Patients With Advanced Solid Tumors	Terminated	No Results Available	Solid Tumor|Tumors of Any Histology With Activating Trk (NTRK) Point or NTRK Fusion Mutations|Tenosynovial Giant Cell Tumor	PLX7486 TsOH	Phase 1	59
15	NCT02829723	A Study of BLZ945 Single Agent or BLZ945 in Combination With PDR001 in Advanced Solid Tumors	Terminated	No Results Available	Advanced Solid Tumors	BLZ945|PDR001	Phase 1|Phase 2	198
16	NCT03557970	JNJ-40346527 in Treating Participants With Relapsed or Refractory Acute Myeloid Leukemia	Terminated	55.0% SD, 40.0% PD. PFS for all treated patients ranged from 2 days to 352+ days.	Recurrent Acute Myeloid Leukemia|Refractory Acute Myeloid Leukemia	Drug: Edicotinib|Other: Pharmacokinetic Study		
17	NCT03177460	Daratumumab or FMS Inhibitor JNJ-40346527 Before Surgery in Treating Patients With High-Risk, Resectable Localized or Locally Advanced Prostate Cancer	Active, not recruiting	No Results Available	Prostate Adenocarcinoma|Stage III Prostate Cancer AJCC v8|Stage IIIA Prostate Cancer AJCC v8|Stage IIIB Prostate Cancer AJCC v8|Stage IIIC Prostate Cancer AJCC v8|Testosterone Greater Than 150 ng/dL	Biological: Daratumumab|Drug: FMS Inhibitor JNJ-40346527|Procedure: Radical Prostatectomy		

##### 3.1.1.1 Pexidartinib (PLX3397, *TURALIO*)

The small molecule drug PLX3397 targets CSF1R signaling and reprograms intra-tumoral immune suppressive myeloid cells (92), and has been shown to convert immune suppressive M-DSCs to a more proinflammatory tumoricidal phenotype (93, 94). PLX3397was approved by the FDA in 2019 for use in the treatment of diffuse type tenosynovial giant cell tumors (dt-TGCT), a rare and often unresectable non-life-threatening cancer of the tendon sheath that is driven by CSF-1 expressing TAMs (95). CSF-1 activation in dt-TGCT leads to recruitment of CSF-1R+ macrophages which make up a large bulk of the tumor mass (96). This specific tumor type is well-suited for targeting by CSF-1/CSF-1R pathway blockade; and treatment with anti-CSF-1R antibodies has shown significant reduction of CSF-1R+ TAMs within tumor tissues (97). In a phase III double blind clinical trial, 14.8% of patients with unresectable dt-TGCT treated with PLX3397 had a complete response (CR) and 24.6% had a partial response (PR) per RECIST criteria compared to zero response in the placebo control group (98).

Current clinical trials are investigating the effectiveness of PLX3397 in multiple cancer types including melanoma, prostate cancer, recurrent glioblastoma multiforme (GBM) and hematological malignancies (99–101). Preclinical use of orally administered PLX3397 for the treatment of recurrent GBM in phase II trials did not show statistically significant improvement in progression free survival of patients compared to historical controls, and there were no partial or complete responses observed in their 38-patient cohort (92, 102). In a phase II trial with 20 patients with relapsed Hodgkin lymphoma, single agent PLX3397 treatment showed an objective overall response rate (ORR) of 5% (103). Thus, the value of CSF-1R inhibition alone for treatment of tumors such as GBM may be limited.

Trials investigating the use of PLX3397 in combination with other agents are ongoing breast cancer (NCT01042379) and unresectable sarcomas and malignant peripheral nerve sheath tumors (NCT02584647). A Phase II trial in patients with advanced melanoma and other solid tumors in combination with PD-1 blocking antibody pembrolizumab (NCT02452424) was terminated early due to insufficient evidence of clinical efficacy (101).

##### 3.1.1.2 Chiauranib (CS2164)

The small molecule drug chiauranib is a CSF-1R inhibitor that also selectively inhibits kinases related to angiogenesis, including VEGF, PDGFR, and c-kit (104). Chiauranib binds to the ATP site in VEGFR2 and inhibits kinase activity, as well as reducing phosphorylation of ERK1/2, thus decreasing expression of genes related to tumor angiogenesis. Chiauranib has shown efficacy in preclinical mouse models of hepatocellular carcinoma, colorectal cancer, and non-Hodgkin lymphoma (NHL) (105–107). Initial dose escalation trials demonstrated that 67% of patients achieved stable disease, with acceptable safety and tolerability (104). Current clinical trials are ongoing, with one phase II trial reported currently in the US (NCT05271292), evaluating chiauranib as a single agent to treat advanced solid malignant tumors.

##### 3.1.1.3 Additional small molecule inhibitors of CSF-1R

Other small molecule CSF-1R inhibitors include ARRY-382, PLX7486, BLZ945 and JNJ-40346527 (edicotinib), and all are currently being evaluated in clinical trials for treatment of Hodgkin lymphoma (cHL) (87). A phase I study with ARRY382 for treatment of advanced solid tumors showed 15% stable disease with no objective responses observed out of 26 patients when administered in combination withpembrolizumab (NCT02880371). Phase I and II clinical studies of the drug JNJ-40346527 in patients with refractory Hodgkin lymphoma found that 11 of 20 patients (55.0%) had stable disease (SD) with progression free survival (PFS) times for all treated patients ranging from 2 days to 352 days (90).

##### 3.1.1.4 Monoclonal antibodies targeting CSF-1 or CSF-R1

Monoclonal antibodies targeting CSF-1R in clinical development include emactuzumab, AMG820, IMC-CS4, cabiralizumab, MCS110 (lacnotuzumab) and PD036324 (**Table 2**). MCS110 and PD036324 target the CSF-1 (ligand) as opposed to the CSF-R1 receptor (87). Phase Ia/Ib trials with emactuzumab as either a single agent or in combination with paclitaxel in patients with metastatic solid tumors including mesothelioma, soft tissue sarcoma, ovarian, breast, pancreatic, endometrial cancer and dt-TGCT have been conducted. Study outcomes in the monotherapy group did not reveal any patients with objective tumor responses, with 13% of patients exhibiting SD. When administered in combination with paclitaxel, 7% of patients had PR with 43% showing SD (108). This study also demonstrated a significant reduction in the numbers of CSF-1R+ TAMs in both monotherapy and combination groups (101). The first human trial of AMG820 showed increased serum CSF-1 concentrations and decreased numbers of macrophages (109). Patients with relapsed or refractory advanced solid tumors treated with AMG820 experienced a 32% SD rate, while one patient with NSCLC experienced a PR. All the agents in trials targeting CSF-R1 have generally been well-tolerated to date, suggesting that sustained CSF-1R blockade treatment for weeks to months is safe. However, to date none of the CSF-1 or CSF-1R targeted agents has demonstrated significant antitumor activity clinically (110).

**Table 2:** US clinical trials in cancer using Monoclonal antibodies targeting CSF-1 or CDF-1R.

CSF-1/CSF-1R monoclonal antibodies	NCT Number	Title	Status	Study Results	Conditions	Interventions	Phases	Enrollment
1	NCT05417789	Study of Emactuzumab for Tenosynovial Giant Cell Tumor (TGCT)	Active, not recruiting	No Results Available	TGCT	Drug: Emactuzumab|Drug: Placebo	Phase 3	128
2	NCT03369964	A Study of Atezolizumab in Combination With an Immunotherapy Agent Investigated With or Without Anti-Cd20 Therapy in Patients With Relapsed or Refractory Non-Hodgkin Lymphoma	Withdrawn	No Results Available	Lymphoma, Non-Hodgkin	Drug: Atezolizumab|Drug: Emactuzumab|Drug: Obinutuzumab	Phase 1	0
3	NCT02760797	A Study of Emactuzumab and RO7009789 Administered in Combination in Participants With Advanced Solid Tumors	Completed	No Results Available	Neoplasms	Drug: Emactuzumab|Drug: RO7009789	Phase 1	38
4	NCT02323191	A Study of Emactuzumab and Atezolizumab Administered in Combination in Participants With Advanced Solid Tumors	Completed	No Results Available	Solid Cancers	Drug: Atezolizumab|Drug: Emactuzumab	Phase 1	221
5	NCT02923739	Paclitaxel and Bevacizumab With or Without Emactuzumab in Treating Patients With Platinum-Resistant Ovarian, Fallopian Tube, or Primary Peritoneal Cancer	Completed	No Results Available	Fallopian Tube Adenocarcinoma|Fallopian Tube Clear Cell Adenocarcinoma|Fallopian Tube Endometrioid Adenocarcinoma|Fallopian Tube Mucinous Adenocarcinoma|Fallopian Tube Serous Adenocarcinoma|Fallopian Tube Transitional Cell Carcinoma|Fallopian Tube Undifferentiated Carcinoma|Malignant Ovarian Brenner Tumor|Ovarian Adenocarcinoma|Ovarian Clear Cell Adenocarcinoma|Ovarian Endometrioid Adenocarcinoma|Ovarian Mucinous Adenocarcinoma|Ovarian Seromucinous Carcinoma|Ovarian Serous Adenocarcinoma|Ovarian Transitional Cell Carcinoma|Ovarian Undifferentiated Carcinoma|Primary Peritoneal Serous Adenocarcinoma|Recurrent Fallopian Tube Carcinoma|Recurrent Ovarian Carcinoma|Recurrent Primary Peritoneal Carcinoma	Biological: Bevacizumab|Biological: Emactuzumab|Other: Laboratory Biomarker Analysis|Drug: Paclitaxel|Other: Pharmacological Study	Phase 2	9
6	NCT01494688	A Study of RO5509554 as Monotherapy and in Combination With Paclitaxel in Participants With Advanced Solid Tumors	Completed	No Results Available	Advanced Solid Tumors	Drug: Paclitaxel|Drug: RO5509554	Phase 1	217
7	NCT01444404	A Study of AMG 820 in Subjects With Advanced Solid Tumors	Completed	No Results Available	Advanced Malignancy|Advanced Solid Tumors	Drug: AMG 820	Phase 1	25
8	NCT02713529	Safety and Efficacy Study of AMG 820 and Pembrolizumab Combination in Select Advanced Solid Tumor Cancer	Completed	Objective Response Rate (ORR) up tp 5.3%, highest OS 75% at. 6 months and 41.7% at 12 months	Pancreatic Cancer|Colorectal Cancer|Non-Small Cell Lung Cancer	Biological: AMG820 and pembrolizumab	Phase 1|Phase 2	117
9	NCT01346358	A Study of IMC-CS4 in Subjects With Advanced Solid Tumors	Completed	No Results Available	Neoplasms	Biological: IMC-CS4	Phase 1	72
10	NCT03153410	Pilot Study With CY, Pembrolizumab, GVAX, and IMC-CS4 (LY3022855) in Patients With Borderline Resectable Adenocarcinoma of the Pancreas	Active, not recruiting	No Results Available	Pancreatic Cancer	Drug: Cyclophosphamide|Drug: GVAX|Drug: Pembrolizumab|Drug: IMC-CS4	Early Phase 1	12
11	NCT02265536	A Study of LY3022855 In Participants With Breast or Prostate Cancer	Completed	No Results Available	Neoplasms|Neoplasm Metastasis	Drug: LY3022855	Phase 1	36
12	NCT03697564	Nivolumab + Cabiralizumab + Gemcitabine in Patients With Stage IV Pancreatic Cancer Achieving Disease Control in Response to First-line Chemotherapy (GemCaN Trial).	Suspended	No Results Available	Pancreatic Cancer Stage IV	Drug: Gemcitabine|Drug: Nivolumab 10 MG/ML Intravenous Solution [OPDIVO]|Drug: Cabiralizumab	Phase 2	40
13	NCT03502330	APX005M With Nivolumab and Cabiralizumab in Advanced Melanoma, Non-small Cell Lung Cancer or Renal Cell Carcinoma	Active, not recruiting	No Results Available	Advanced Melanoma|Non-small Cell Lung Cancer|Renal Cell Carcinoma	Drug: APX005M|Drug: Cabiralizumab|Drug: Nivolumab	Phase 1	42
14	NCT04848116	Neoadjuvant Targeting of Myeloid Cell Populations in Combination With Nivolumab in Head & Neck Cancer	Recruiting	No Results Available	Head and Neck Squamous Cell Carcinoma	Drug: Nivolumab|Drug: HuMax-IL8|Drug: Cabiralizumab	Phase 2	24
15	NCT03927105	Nivolumab and the Antagonistic CSF-1R Monoclonal Antibody Cabiralizumab (BMS-986227) in Patients With Relapsed/Refractory Peripheral T Cell Lymphoma	Active, not recruiting	2 paitients 4 month CR, 1NR,	Peripheral T Cell Lymphoma	Drug: Nivolumab|Drug: cabiralizumab	Phase 2	4
16	NCT03431948	Stereotactic Body Radiotherapy (SBRT) Plus Immunotherapy for Cancer	Completed	No Results Available	Cancer	Drug: Nivolumab|Drug: Cabiralizumab|Drug: Urelumab|Radiation: Stereotactic Body Radiation Therapy	Phase 1	60
17	NCT04050462	Nivolumab Combined With BMS-986253 in HCC Patients	Active, not recruiting	No Results Available	Hepatocellular Carcinoma	Drug: Nivolumab 240 mg IV every 2 weeks + Cabiralizumab 4 mg/kg IV every 2 weeks|Drug: Nivolumab 240 mg IV every 2 weeks + BMS-986253 1200 mg IV every 2 weeks|Drug: Nivolumab 240 mg IV every 2 weeks	Phase 2	23
18	NCT04331067	Neoadjuvant Nivolumab and Chemotherapy in Patients With Localized Triple-negative Breast Cancer	Recruiting	No Results Available	Triple Negative Breast Cancer	Drug: Paclitaxel|Drug: Carboplatin|Biological: Nivolumab|Biological: Cabiralizumab|Procedure: Tumor biopsy|Procedure: Bone marrow|Procedure: Blood draw	Phase 1|Phase 2	31
19	NCT02526017	Study of Cabiralizumab in Combination With Nivolumab in Patients With Selected Advanced Cancers	Completed	highest OS group 13 months , highest PFS 2.9 months	Advanced Solid Tumors|Head and Neck Cancer|Pancreatic Cancer|Ovarian Cancer|Renal Cell Carcinoma|Malignant Glioma|Non-small Cell Lung Cancer	Biological: Cabiralizumab|Biological: Nivolumab	Phase 1	313
20	NCT02471716	Study of Cabiralizumab in Patients With Pigmented Villonodular Synovitis / Diffuse Type Tenosynovial Giant Cell Tumor	Completed	ORR up to 33%	Pigmented Villonodular Synovitis|Tenosynovial Giant Cell Tumor	Biological: FPA008	Phase 1|Phase 2	66
21	NCT03336216	A Study of Cabiralizumab Given With Nivolumab With and Without Chemotherapy in Patients With Advanced Pancreatic Cancer	Active, not recruiting	No Results Available	Advanced Pancreatic Cancer	Biological: Cabiralizumab|Drug: Nab-paclitaxel|Drug: Onivyde|Biological: Nivolumab|Drug: Fluorouracil|Drug: Gemcitabine|Drug: Oxaliplatin|Drug: Leucovorin|Drug: Irinotecan Hydrochloride	Phase 2	202
22	NCT03335540	An Adaptive Study to Match Patients With Solid Tumors to Various Immunotherapy Combinations Based Upon a Broad Biomarker Assessment	Completed	No Results Available	Advanced Cancer	Biological: Nivolumab|Biological: Relatlimab|Biological: Cabiralizumab|Biological: Ipilimumab|Drug: IDO1 Inhibitor|Radiation: Radiation Therapy	Phase 1	20
23	NCT03455764	MCS110 With BRAF/MEK Inhibition in Patients With Melanoma	Active, not recruiting	No Results Available	Melanoma	Drug: MCS110|Drug: Dabrafenib|Drug: Trametinib	Phase 1|Phase 2	43
24	NCT01643850	MCS110 in Patients With Pigmented Villonodular Synovitis (PVNS)	Completed	decrease in tumor size	Pigmented Villonodular Synovitis|PVNS|Giant Cell Tumor of the Tendon Sheath|GCCTS|Tenosynovial Giant Cell Tumor Localized or Diffused Type|GCTS	Drug: MCS110|Drug: Placebo	Phase 2	36
25	NCT02807844	Phase Ib/II Study of MCS110 in Combination With PDR001 in Patients With Advanced Malignancies	Completed	Clinical Benefit Rate up to 20%	Triple Negative Breast Cancer|Pancreatic Carcinoma|Melanoma|Endometrial Carcinoma	Drug: MCS110|Drug: PDR001	Phase 1|Phase 2	141
26	NCT02435680	Efficacy Study of MCS110 Given With Carboplatin and Gemcitabine in Advanced Triple Negative Breast Cancer (TNBC)	Completed	PFS average 5.6 months, SD up tp 55.9%, ORR up to 37.5%	Advanced Triple Negative Breast Cancer (TNBC) With High TAMs	Drug: MCS110|Drug: carboplatin|Drug: gemcitabine	Phase 2	50
27	NCT03742349	Study of Safety and Efficacy of Novel Immunotherapy Combinations in Patients With Triple Negative Breast Cancer (TNBC).	Active, not recruiting	No Results Available	Triple Negative Breast Cancer (TNBC)	Biological: spartalizumab|Biological: LAG525|Drug: NIR178|Drug: capmatinib|Biological: MCS110|Biological: canakinumab	Phase 1	64
29	NCT02554812	A Study Of Avelumab In Combination With Other Cancer Immunotherapies In Advanced Malignancies (JAVELIN Medley)	Active, not recruiting	No Results Available	Advanced Cancer	Drug: Avelumab|Drug: Utomilumab|Drug: PF-04518600|Drug: PD 0360324|Drug: CMP-001	Phase 1|Phase 2	398

#### 3.1.2 Trabectedin as myeloid cell depleting chemotherapy

Trabectedin an alkaloid drug that binds a minor groove of DNA and blocks the cell cycle and DNA repair pathways (111). It has been shown to selectively reduce TAMs in tumors without affecting the infiltration of T cells (112). Treatment with trabectedin also inhibits local differentiation of monocytes into TAMs (113). Use of trabectedin in multiple preclinical animal tumor models demonstrated depletion of TAMs and reduction of tumor growth, suppression of angiogenesis, and reduced concentrations of IL6, CCL2 and CXCL8 (114). Current phase II clinical trials of trabectedin are ongoing for treatment of soft tissue sarcoma, bone tumors and small round-cell sarcomas, administered in combination with low-dose radiation therapy (NCT05131386). Trabectedin is FDA approved for treatment of unresectable or metastatic liposarcoma and leiomyosarcoma (115) (**Table 3**).

**Table 3:** US clinical trials using Trabectedin as myeloid cell depleting chemotherapy for cancer.

**Myeloid** **targeted** **chemotherapy**	**NCT Number**	**Title**	**Status**	**Study Results**	**Conditions**	**Interventions**	**Phases**	**Enrollment**
1	NCT03886311	Talimogene Laherparepvec, Nivolumab and Trabectedin for Sarcoma	Recruiting	No Results Available	Sarcoma	Drug: Talimogene Laherparepvec 100000000 PFU/1 ML Injection Suspension [IMLYGIC]|Drug: Nivolumab IV Soln 100 MG/10ML|Drug: Trabectedin 0.25 MG/1 VIAL Intravenous Powder for Solution	Phase 2	40
2	NCT04535271	Metronomic Trabectedin, Gemcitabine, and Dacarbazine for Leiomyosarcoma	Recruiting	No Results Available	Leiomyosarcoma	Drug: Trabectedin	Phase 2	80
3	NCT04076579	Trabectedin in Combination With Olaparib in Advanced Unresectable or Metastatic Sarcoma	Active, not recruiting	No Results Available	Sarcoma|Sarcoma Metastatic	Drug: Olaparib|Drug: Trabectedin	Phase 2	29
4	NCT00072670	A Phase 2 Study of Trabectedin (Yondelis) in Adult Male Participants With Advanced Prostate Cancer	Completed	No Results Available	Prostate Cancer	Drug: Trabectedin	Phase 2	59
5	NCT03074318	Avelumab and Trabectedin in Treating Patients With Liposarcoma or Leiomyosarcoma That is Metastatic or Cannot Be Removed by Surgery	Terminated	up to 18.8% PR , 66.7% SD at 12 weeks, clinical benefit rate 56%. OS highest group average 416 days	Metastatic Leiomyosarcoma|Metastatic Liposarcoma|Unresectable Leiomyosarcoma|Unresectable Liposarcoma	Drug: Avelumab|Drug: Trabectedin	Phase 1|Phase 2	35
6	NCT03138161	SAINT:Trabectedin, Ipilimumab and Nivolumab as First Line Treatment for Advanced Soft Tissue Sarcoma	Recruiting	No Results Available	Advanced Soft Tissue Sarcoma|Metastatic Soft Tissue Sarcoma	Drug: Trabectedin|Drug: Ipilimumab|Drug: Nivolumab	Phase 1|Phase 2	45
7	NCT00147212	ET 743 (Yondelis) in Men With Advanced Prostate Cancer	Completed	Prostate specific antigen (PSA) response rate 7/50	Prostate Cancer	Drug: ET 743	Phase 2	50

### 3.2 Chemokine receptor antagonists for monocyte and neutrophil migration inhibition

Chemokine receptor antagonists can reduce the infiltration of monocytes and MDSCs into the TME. The chemokine CCL2 binds to the receptor CCR2 expressed on inflammatory monocytes (116), which signals to circulating monocytes to promote extravasation from the vasculature and into inflamed tissues (32). Many tumors secrete large amounts of CCL2, thereby recruiting circulating inflammatory monocytes into tumor tissues where they then differentiate into M2 TAMs (32, 117, 118). CCL2 may also play a minor role in PMN-MDSC recruitment, though the primary chemokine driving TAN recruitment is CXCL8 (119). There have been numerous preclinical studies in rodent models assessing inhibitors of the CCL2-CCR2 axis using either small molecule CCL2 inhibitors or monoclonal antibodies, and most have demonstrated inhibition of tumor growth and/or decreased metastatic burden (120). In these models, CCL2-CCR2 signaling blockade has been shown to suppress tumor growth through multiple pathways including depletion of TAMs and M-MDSC and increasing infiltrating T cells (32, 118, 120).

#### 3.2.1 CCR2 targeted antibodies

Carlumab (CNTO888) is a CCL2 neutralizing antibody that has been evaluated in multiple cancer models as either a single agent immunotherapy or in combination with chemotherapy (121). Pre-clinical mouse models evaluating carlumab have demonstrated increased IFNγ production by NK cells and antitumoral CD8+ T cells when combined with anticancer vaccines (122). Carlumab has demonstrated positive clinical responses when used in combination with chemotherapeutic drug docetaxel (123); phase II trials have been completed but Carlumab has since been discontinued (NCT00992186).

MLN1202 (plozalizumab) is a CCR2 blocking monoclonal antibody currently undergoing phase II clinical trials for treatment of metastatic bone cancer (NCT01015560). Results so far show that MLN1202 is relatively well tolerated with only 7.14% of patients experiencing severe adverse events (SAE). A phase I trial of MLN1202 in combination with nivolumab was terminated early due to serious adverse events (NCT02723006), which may suggest limited potential for MLN1202 as single or combined immunotherapy agent (124, 125) (**Table 4**).

**Table 4:** US clinical trials in cancer targeting CCR2 or CCR5 axis.

CCL2/CCR2 blockade	NCT Number	Title	Status	Study Results	Conditions	Interventions	Phases	Enrollment
1	NCT01015560	S0916, MLN1202 in Treating Patients With Bone Metastases	Completed	7.14% SAE	Metastatic Cancer|Unspecified Adult Solid Tumor, Protocol Specific	Drug: anti-CCR2 monoclonal antibody MLN1202|Genetic: polymorphism analysis|Other: laboratory biomarker analysis	Phase 2	44
2	NCT02723006	Study to Evaluate the Safety, Tolerability, and Pharmacodynamics of Investigational Treatments in Combination With Standard of Care Immune Checkpoint Inhibitors in Participants With Advanced Melanoma	Terminated	up to 58.33% in arm 3 triple drug combo	Melanoma	Drug: TAK-580|Drug: TAK-202|Drug: vedolizumab|Drug: nivolumab|Drug: ipilimumab	Phase 1	22
3	NCT01413022	FOLFIRINOX Plus PF-04136309 in Patients With Borderline Resectable and Locally Advanced Pancreatic Adenocarcinoma	Completed	No Results Available	Pancreatic Neoplasms	Drug: Oxaliplatin|Drug: Irinotecan|Drug: Leucovorin|Drug: Fluorouracil|Other: laboratory biomarker analysis|Other: flow cytometry|Other: immunohistochemistry staining method|Other: pharmacological study|Drug: PF-04136309	Phase 1	44
4	NCT02732938	Ph1b/2 Study of PF-04136309 in Combination With Gem/Nab-P in First-line Metastatic Pancreatic Patients	Terminated	PFS not reported, 11/17 SAE in arm 1b combination treatment	Metastatic Pancreatic Ductal Adenocarcinoma	Drug: PF-04136309|Drug: Nab-paclitaxel|Drug: Gemcitabine	Phase 2	22
CCR2/CCR5 blockade	NCT Number	Title	Status	Study Results	Conditions	Interventions	Phases	Enrollment
1	NCT03184870	A Study of BMS-813160 in Combination With Chemotherapy or Nivolumab in Participants With Advanced Solid Tumors	Active, not recruiting	No Results Available	Colorectal Cancer|Pancreatic Cancer	Drug: BMS-813160|Biological: Nivolumab|Drug: Nab-paclitaxel|Drug: Gemcitabine|Drug: 5-fluorouracil (5-FU)|Drug: Leucovorin|Drug: Irinotecan	Phase 1|Phase 2	332
2	NCT04123379	Neoadjuvant Nivolumab With CCR2/5-inhibitor or Anti-IL-8) for Non-small Cell Lung Cancer (NSCLC) or Hepatocellular Carcinoma (HCC)	Recruiting	No Results Available	Non-small Cell Lung Cancer|Hepatocellular Carcinoma	Drug: Nivolumab|Drug: BMS-813160|Drug: BMS-986253	Phase 2	50
3	NCT02996110	A Study to Test Combination Treatments in People With Advanced Renal Cell Carcinoma	Completed	ORR up tp 17.4%, PFS at 24 Weeks up to 46.8% in arm5	Advanced Cancer	Biological: Nivolumab|Biological: Ipilimumab|Biological: Relatlimab|Drug: BMS-986205|Drug: BMS-813160	Phase 2	182
4	NCT03767582	Trial of Neoadjuvant and Adjuvant Nivolumab and BMS-813160 With or Without GVAX for Locally Advanced Pancreatic Ductal Adenocarcinomas.	Recruiting	No Results Available	Locally Advanced Pancreatic Ductal Adenocarcinoma (PDAC)|Pancreatic Ductal Adenocarcinoma	Radiation: Stereotactic Body Radiation (SBRT)|Drug: Nivolumab|Drug: CCR2/CCR5 dual antagonist|Drug: GVAX	Phase 1|Phase 2	30
5	NCT03496662	BMS-813160 With Nivolumab and Gemcitabine and Nab-paclitaxel in Borderline Resectable and Locally Advanced Pancreatic Ductal Adenocarcinoma (PDAC)	Active, not recruiting	SAE up to 68.00% in dose expantion	Pancreatic Ductal Adenocarcinoma	Drug: BMS-813160|Drug: Nivolumab|Drug: Gemcitabine|Drug: Nab-paclitaxel|Procedure: Biopsy|Procedure: Peripheral blood	Phase 1|Phase 2	40

#### 3.2.2 CCL2 inhibitors

Bindarit is a small molecule drug that inhibits the synthesis of CCL2 and has been shown to induce tumor regression in preclinical studies by inhibiting TAM and MDSC infiltration of the TME in breast cancer, prostate cancer, and osteosarcoma animal models (126–129). A second CCL2 inhibitor mNOX-36 has been shown in a rat model of GBM to significantly inhibit tumor growth (130). The safety of mNOX-36 is currently being evaluated in Phase I trials (**Table 4**).

#### 3.2.3 CCR2 inhibitors

RS 504393 is a small molecule CCR2 antagonist that has shown activity in blocking M-MDSCs and TAM recruitment into tumors following gemcitabine treatment in a mouse model of bladder cancer (131). Another CCR2 inhibitor (BMS CCR2 22), is a high affinity CCR2 antagonist that decreases TAM density as demonstrated in mouse metastatic hepatic cancer models. When combined with FOLFOX (folinic acid, fluorouracil oxaliplatin) chemotherapy regimine, administration of BMS CCR2 22 significantly increased efficacy and improved overall survival in mice with colon adenocarcinomas (117, 118). A third CCR2 antagonist, 747 is a natural product derived from the tree *Abies georgei* (132). The drug 747 is considered a selective CCR2 antagonist and has been shown to inhibit TAM recruitment and increase density of CD8+ tumor infiltrating lymphocytes as well as increase inflammatory cytokines such as IFN-γ in rodent mode. Treatment with 747 also increased tumor apoptosis when combined with sorafenib, a tyrosine kinase inhibitor, thereby potentiating antitumor activity by depleting TAMs (133).

A fourth selective CCR2 inhibitor (PF-04136309) has demonstrated antitumor activity in an orthotopic mouse model of pancreatic cancer (134). Phase Ib clinical trials in patients with pancreatic cancer evaluated treatment with PF-04136309 in combination with the chemotherapy regimen FOLFIRINOX (folinic acid, fluorouracil, irinotecan hydrochloride, and oxaliplatin) and demonstrated a 49% response rate, compare to no responding patients treated with FOLFIRINOX alone. In addition, administration of PF-04136309 in combination with FOLFIRINOX significantly decreased the numbers of CCR2+ monocytes in bone marrow samples, compared to FOLFIRINOX alone treated patients (135). A phase I study in patients with metastatic pancreatic cancer found that PF-04136309 given in combination with chemotherapy gemcitabine and nab-paclitaxel significantly decreased CD14+CCR2+ monocytes in circulation. However, the high incidence of pulmonary toxicity in patients treated with PF-04136309 led to a discontinuation of further clinical evaluation (136).

#### 3.2.4 Dual CCR2/CCR5 inhibitor for myeloid cell targeting

BMS-813160 is a dual CCR2/CCR5 inhibitor which has been investigated in phase I and phase II trials as combination therapy (137). In ongoing phase II clinical trials for treatment of NSCLC and hepatocellular cancer, BMS-813160 is being administered in combination with nivolumab and the anti-CXCL8 drug BMS-986253 (NCT04123379) (138). BMS-813260 is also being investigated in phase II trials for pancreatic ductal carcinoma and colorectal cancer, administered in combination with either nivolumab or chemotherapy (139) (**Table 4**).

#### 3.2.5 Repurposed angiotensin receptor antagonists for CCR2 inhibition

Losartan, a type 1 angiotensin II receptor (AT1R) blocker (ARB), has been found to exert off-target activity as a potent, non-competitive CCR2 antagonist (140). In a mouse syngeneic breast cancer model, losartan suppressed lung metastatic tumor burden significantly (141). In this model, the reduced metastatic burden was associated with a significant decrease in CD11b+/Ly6C+ monocytes recruited to the lungs (140). In studies in a dog model of metastatic osteosarcoma, the combination high dose losartan (10mg/kg PO BID) with the non-selective tyrosine kinase inhibitor toceranib demonstrated a response rate (PR) of 25% and clinical benefit rate of 50% (142). A similar phase I clinical trial is underway for pediatric osteosarcoma using the combination of losartan with the non-selective tyrosine kinase inhibitor sunitinib (NCT03900793). There are also multiple other clinical trials currently evaluating losartan in combination with radiation therapy and chemotherapy or immunotherapy. A phase II clinical trial of losartan in combination with nivolumab is currently underway in patients with localized pancreatic cancer (NCT03563248). In addition, losartan is being evaluated in combination with radiation therapy and chemotherapy in pancreatic cancer (NCT03563248, NCT04106856). A recent study also indicates that losartan treatment can reduce cerebral edema following immunotherapy in a rodent GBM model (143) (**Table 5**).

**Table 5:** US clinical trials using Repurposed angiotensin receptor antagonists for CCR2 inhibition in cancer.

Losartan CCL2 blockade	NCT Number	Title	Status	Study Results	Conditions	Interventions	Phases	Enrollment
1	NCT01821729	Proton w/FOLFIRINOX-Losartan for Pancreatic Cancer	Unknown status	SAE 30.61%	Pancreatic Cancer	Drug: FOLFIRINOX|Drug: Losartan|Radiation: Proton Beam Radiation	Phase 2	50
2	NCT04106856	Losartan and Hypofractionated Rx After Chemo for Tx of Borderline Resectable or Locally Advanced Unresectable Pancreatic Cancer (SHAPER)	Recruiting	No Results Available	Borderline Resectable Pancreatic Adenocarcinoma|Locally Advanced Pancreatic Ductal Adenocarcinoma|Locally Advanced Unresectable Pancreatic Adenocarcinoma|Stage II Pancreatic Cancer AJCC v8|Stage IIA Pancreatic Cancer AJCC v8|Stage IIB Pancreatic Cancer AJCC v8|Stage III Pancreatic Cancer AJCC v8	Radiation: Hypofractionated Radiation Therapy|Drug: Losartan|Drug: Losartan Potassium|Other: Quality-of-Life Assessment|Other: Questionnaire Administration	Phase 1	20
3	NCT05077800	FOLFIRINOX + 9-Ing-41 + Losartan In Pancreatic Cancer	Recruiting	No Results Available	Pancreatic Adenocarcinoma|Pancreatic Adenocarcinoma Metastatic	Drug: FOLFIRNINOX|Drug: Losartan|Drug: 9-ING-41	Phase 2	70
4	NCT05365893	PHL Treatment in Pancreatic Cancer	Recruiting	No Results Available	Pancreatic Ductal Adenocarcinoma	Combination Product: Paricalcitol, Hydroxychloroquine, Losartan|Other: Neoadjuvant therapy and surgery only (Control)	Early Phase 1	20
5	NCT01234922	Benazepril Hydrochloride, Lisinopril, Ramipril, or Losartan Potassium in Treating Hypertension in Patients With Solid Tumors	Terminated	Protocol was closed early due to slow accrual, no SAE observed	Hypertension|Unspecified Adult Solid Tumor, Protocol Specific	Drug: lisinopril|Drug: losartan potassium|Other: laboratory biomarker analysis|Drug: benazepril hydrochloride|Drug: ramipril	Phase 2	6
6	NCT01276613	Tissue Pharmacokinetics of Intraoperative Gemcitabine in Resectable Adenocarcinoma of the Pancreas	Terminated	No Results Available	Pancreatic Cancer	Drug: Gemcitabine|Drug: Losartan	Early Phase 1	18
7	NCT04539808	NeoOPTIMIZE: Early Switching of mFOLFIRINOX or Gemcitabine/Nab-Paclitaxel Before Surgery for the Treatment of Resectable, Borderline Resectable, or Locally-Advanced Unresectable Pancreatic Cancer	Recruiting	No Results Available	Borderline Resectable Pancreatic Carcinoma|Locally Advanced Unresectable Pancreatic Adenocarcinoma|Resectable Pancreatic Ductal Adenocarcinoma|Stage 0 Pancreatic Cancer AJCC v8|Stage I Pancreatic Cancer AJCC v8|Stage IA Pancreatic Cancer AJCC v8|Stage IB Pancreatic Cancer AJCC v8|Stage III Pancreatic Cancer AJCC v8|Stage IV Pancreatic Cancer AJCC v8	Drug: Capecitabine|Drug: Fluorouracil|Drug: Irinotecan Hydrochloride|Drug: Leucovorin Calcium|Drug: Losartan Potassium|Drug: Oxaliplatin|Radiation: Radiation Therapy|Procedure: Resection	Phase 2	60
8	NCT05607017	Losartan in Prevention of Radiation-Induced Heart Failure	Not yet recruiting	No Results Available	Breast Cancer|Myocardial Fibrosis|Radiation-Induced Fibrosis	Drug: Losartan|Radiation: Radiation Therapy	Early Phase 1	10
9	NCT03563248	Losartan and Nivolumab in Combination With FOLFIRINOX and SBRT in Localized Pancreatic Cancer	Active, not recruiting	No Results Available	Pancreatic Cancer	Drug: FOLFIRINOX|Drug: Losartan|Drug: Nivolumab|Radiation: SBRT|Procedure: Surgery	Phase 2	168
10	NCT03864042	Pharmacokinetic Drug-drug Interaction Study of Encorafenib and Binimetinib on Probe Drugs in Patients With BRAF V600-mutant Melanoma or Other Advanced Solid Tumors	Active, not recruiting	No Results Available	Advanced Solid Tumors|Metastatic Melanoma	Drug: losartan|Drug: dextromethorphan|Drug: caffeine|Drug: omeprazole|Drug: midazolam|Drug: rosuvastatin|Drug: bupropion immediate release (IR)|Drug: encorafenib|Drug: binimetinib|Drug: modafinil	Phase 1	56
11	NCT03900793	Losartan + Sunitinib in Treatment of Osteosarcoma	Recruiting	No Results Available	Osteosarcoma	Drug: Losartan|Drug: Sunitinib	Phase 1	41
12	NCT01199978	Hearing Outcomes Using Fractionated Proton Radiation Therapy for Vestibular Schwannoma	Active, not recruiting	No Results Available	Vestibular Schwannoma|Acoustic Neuroma	Radiation: Fractionated proton radiation|Drug: Losartan	Phase 2	30
13	NCT03878524	Serial Measurements of Molecular and Architectural Responses to Therapy (SMMART) PRIME Trial	Recruiting	No Results Available	Accelerated Phase Chronic Myelogenous Leukemia, BCR-ABL1 Positive|Anatomic Stage IV Breast Cancer AJCC v8|Anemia|Ann Arbor Stage III Hodgkin Lymphoma|Ann Arbor Stage III Non-Hodgkin Lymphoma|Ann Arbor Stage IV Hodgkin Lymphoma|Ann Arbor Stage IV Non-Hodgkin Lymphoma|Atypical Chronic Myeloid Leukemia, BCR-ABL1 Negative|Blast Phase Chronic Myelogenous Leukemia, BCR-ABL1 Positive|Castration-Resistant Prostate Carcinoma|Chronic Phase Chronic Myelogenous Leukemia, BCR-ABL1 Positive|Hematopoietic and Lymphoid System Neoplasm|Locally Advanced Pancreatic Adenocarcinoma|Metastatic Breast Carcinoma|Metastatic Malignant Solid Neoplasm|Metastatic Pancreatic Adenocarcinoma|Myelodysplastic/Myeloproliferative Neoplasm With Ring Sideroblasts and Thrombocytosis|Myelodysplastic/Myeloproliferative Neoplasm, Unclassifiable|Primary Myelofibrosis|Recurrent Acute Lymphoblastic Leukemia|Recurrent Acute Myeloid Leukemia|Recurrent Chronic Lymphocytic Leukemia|Recurrent Chronic Myelogenous Leukemia, BCR-ABL1 Positive|Recurrent Hematologic Malignancy|Recurrent Hodgkin Lymphoma|Recurrent Myelodysplastic Syndrome|Recurrent Myelodysplastic/Myeloproliferative Neoplasm|Recurrent Myeloproliferative Neoplasm|Recurrent Non-Hodgkin Lymphoma|Recurrent Plasma Cell Myeloma|Recurrent Small Lymphocytic Lymphoma|Refractory Acute Lymphoblastic Leukemia|Refractory Acute Myeloid Leukemia|Refractory Chronic Lymphocytic Leukemia|Refractory Chronic Myelogenous Leukemia, BCR-ABL1 Positive|Refractory Chronic Myelomonocytic Leukemia|Refractory Hematologic Malignancy|Refractory Hodgkin Lymphoma|Refractory Malignant Solid Neoplasm|Refractory Myelodysplastic Syndrome|Refractory Myelodysplastic/Myeloproliferative Neoplasm|Refractory Non-Hodgkin Lymphoma|Refractory Plasma Cell Myeloma|Refractory Primary Myelofibrosis|Refractory Small Lymphocytic Lymphoma|Stage II Pancreatic Cancer AJCC v8|Stage III Pancreatic Cancer AJCC v8|Stage IV Pancreatic Cancer AJCC v8|Stage IV Prostate Cancer AJCC v8|Unresectable Pancreatic Adenocarcinoma	Drug: Abemaciclib|Drug: Abiraterone|Drug: Afatinib|Biological: Bevacizumab|Drug: Bicalutamide|Procedure: Biospecimen Collection|Drug: Bortezomib|Drug: Cabazitaxel|Drug: Cabozantinib|Drug: Capecitabine|Drug: Carboplatin|Drug: Celecoxib|Drug: Cobimetinib|Drug: Copanlisib|Drug: Dabrafenib|Drug: Dacomitinib|Drug: Darolutamide|Drug: Dasatinib|Drug: Doxorubicin|Biological: Durvalumab|Drug: Enasidenib|Drug: Entrectinib|Drug: Enzalutamide|Drug: Erlotinib|Drug: Everolimus|Drug: Fluorouracil|Drug: Idelalisib|Drug: Imatinib|Biological: Ipilimumab|Drug: Lenvatinib|Drug: Leucovorin|Drug: Lorlatinib|Drug: Losartan|Drug: Nab-paclitaxel|Drug: Neratinib|Biological: Nivolumab|Drug: Olaparib|Drug: Oxaliplatin|Drug: Palbociclib|Drug: Panobinostat|Biological: Pembrolizumab|Biological: Pertuzumab|Drug: Ponatinib|Other: Quality-of-Life Assessment|Drug: Regorafenib|Drug: Ruxolitinib|Drug: Sirolimus|Drug: Sorafenib|Drug: Sunitinib|Drug: Trametinib|Biological: Trastuzumab Emtansine|Drug: Tretinoin|Drug: Vemurafenib|Drug: Venetoclax|Drug: Vismodegib|Drug: Vorinostat	Phase 1	40

#### 3.2.6 CXCR1 blockade to deplete PMN-MDSC and TANs

The chemokine CXCL8, which signals *via* CXCR1 and CXCR2, triggers the recruitment of PMN-MDSC and TANs into the TME (144). The receptors CXCR1 and CXCR2 are primarily expressed on neutrophils (145). CXCR1 is very selective for CXCL8, whereas CXCR2 also binds other chemokines. Signaling by CXCR1 and CXCR2 are major mechanisms for recruiting neutrophils and PMN-MDSC into the TME which then differentiate into TANs or PMN-MDSCs (146). High expression of CXCL8 by tumors has been correlated with poor prognosis in many tumor types (147). Thus, CXCR1 and CXCR2 antagonists have been evaluated as strategies to deplete the TME of immune suppressive N2 TANs and PMN-MDSC (48, 148) (**Table 6**).

**Table 6:** US clinical trials using CXCR1 antagonists for cancer treatment.

CXCR1/2 blockade	NCT Number	Title	Status	Study Results	Conditions	Interventions	Phases	Enrollment
Navarixin	NCT03473925	Efficacy and Safety Study of Navarixin (MK-7123) in Combination With Pembrolizumab (MK-3475) in Adults With Selected Advanced/Metastatic Solid Tumors (MK-7123-034)	Completed	ORR up to 3.9%, PFS up to 17.5 mo in NSCLC, OS up to 13.0 mo.	Solid Tumors|Non-small Cell Lung Cancer|Castration Resistant Prostate Cancer|Microsatellite Stable Colorectal Cancer	Drug: Navarixin|Biological: Pembrolizumab	Phase 2	107
Reparixin	NCT02001974	Pilot Study to Evaluate Reparixin With Weekly Paclitaxel in Patients With HER 2 Negative Metastatic Breast Cancer (MBC)	Completed	Clinical Benefit Rate (CBR) up to 56.5% in group 3 combination treatment. 6mo PFS 25.0%.	Metastatic Breast Cancer	Drug: Paclitaxel+Reparixin	Phase 1	33
	NCT01861054	Pilot Study to Evaluate Safety & Biological Effects of Orally Administered Reparixin in Early Breast Cancer	Terminated	5% SAE due to post op infection	Breast Cancer	Drug: Reparixin	Phase 2	20
	NCT02370238	A Double-blind Study of Paclitaxel in Combination With Reparixin or Placebo for Metastatic Triple-Negative Breast Cancer	Completed	non placebo group SD 15/57, CR 1, PR 15/57	Metastatic Breast Cancer	Drug: paclitaxel|Drug: Reparixin|Drug: placebo	Phase 2	194
AZD5069	NCT02499328	Study to Assess MEDI4736 With Either AZD9150 or AZD5069 in Advanced Solid Tumors & Relapsed Metastatic Squamous Cell Carcinoma of Head & Neck	Active, not recruiting	SAE 0 ~ 64.29% in dose escalation	Advanced Solid Tumors & Metastatic Squamous Cell Carcinoma of the Head and Neck	Drug: AZD9150|Drug: MEDI4736|Drug: AZD5069|Drug: tremelimumab (treme)	Phase 1|Phase 2	340
	NCT02583477	Phase Ib/II Study of MEDI4736 Evaluated in Different Combinations in Metastatic Pancreatic Ductal Carcinoma	Completed	Dose-Limiting Toxicities (DLT) up to 33.3%, SAE up to 80.00% in cohort 2, study was terminated by sponsor	Metastatic Pancreatic Ductal Adenocarcinoma	Drug: MEDI4736 in combination with nab-paclitaxel and gemcitabine|Drug: MEDI4736 in combination with AZD5069	Phase 1|Phase 2	23
SX-682	NCT05604560	A Neoadjuvant Study of Tislelizumab and SX-682 for Resectable Pancreas Cancer	Not yet recruiting	No Results Available	Pancreatic Cancer	Drug: Tislelizumab|Drug: SX-682	Phase 2	25
	NCT04574583	Phase I/II Trial Investigating the Safety, Tolerability, Pharmacokinetics, Immune and Clinical Activity of SX-682 in Combination With BinTrafusp Alfa (M7824 or TGF-beta "Trap"/PD-L1) With CV301 TRICOM in Advanced Solid Tumors (STAT)	Active, not recruiting	No Results Available	Metastatic Cancer|Solid Tumors	Drug: SX-682|Drug: M7824|Biological: MVA-BN-CV301|Biological: FPV-CV301	Phase 1|Phase 2	12
	NCT05570825	SX-682 With Pembrolizumab for the Treatment of Metastatic or Recurrent Stage IIIC or IV Non-Small Cell Lung Cancer	Recruiting	No Results Available	Metastatic Lung Non-Small Cell Carcinoma|Recurrent Lung Non-Small Cell Carcinoma|Stage IIIC Lung Cancer AJCC v8|Stage IV Lung Cancer AJCC v8	Procedure: Biopsy|Procedure: Biospecimen Collection|Procedure: Computed Tomography|Drug: CXCR1/2 Inhibitor SX-682|Procedure: Magnetic Resonance Imaging|Biological: Pembrolizumab|Procedure: Positron Emission Tomography	Phase 2	30
	NCT04599140	SX-682 and Nivolumab for the Treatment of RAS-Mutated, MSS Unresectable or Metastatic Colorectal Cancer, the STOPTRAFFIC-1 Trial	Recruiting	No Results Available	Metastatic Colon Adenocarcinoma|Metastatic Colorectal Carcinoma|Metastatic Rectal Adenocarcinoma|Stage III Colon Cancer AJCC v8|Stage III Rectal Cancer AJCC v8|Stage IIIA Colon Cancer AJCC v8|Stage IIIA Rectal Cancer AJCC v8|Stage IIIB Colon Cancer AJCC v8|Stage IIIB Rectal Cancer AJCC v8|Stage IIIC Colon Cancer AJCC v8|Stage IIIC Rectal Cancer AJCC v8|Stage IV Colon Cancer AJCC v8|Stage IV Rectal Cancer AJCC v8|Stage IVA Colon Cancer AJCC v8|Stage IVA Rectal Cancer AJCC v8|Stage IVB Colon Cancer AJCC v8|Stage IVB Rectal Cancer AJCC v8|Stage IVC Colon Cancer AJCC v8|Stage IVC Rectal Cancer AJCC v8|Unresectable Colon Adenocarcinoma|Unresectable Rectal Adenocarcinoma	Drug: CXCR1/2 Inhibitor SX-682|Biological: Nivolumab	Phase 1|Phase 2	53
	NCT04477343	A Study to Evaluate the Safety and Tolerability of SX-682 in Combination With Nivolumab as a Maintenance Therapy in Patients With Metastatic Pancreatic Ductal Adenocarcinoma	Recruiting	No Results Available	Pancreatic Ductal Adenocarcinoma|Pancreatic Cancer	Drug: SX-682|Drug: Nivolumab Injectable Product	Phase 1	20
	NCT03161431	SX-682 Treatment in Subjects With Metastatic Melanoma Concurrently Treated With Pembrolizumab	Recruiting	No Results Available	Melanoma Stage III|Melanoma Stage IV	Drug: SX-682|Biological: Pembrolizumab	Phase 1	77
	NCT04245397	SX-682 Treatment in Subjects With Myelodysplastic Syndrome Who Had Disease Progression or Are Intolerant to Prior Therapy	Recruiting	No Results Available	Myelodysplastic Syndromes	Drug: SX-682	Phase 1	64

##### 3.2.6.1 CXCR1 antagonist navarixin

The selective CXCR1 antagonist navarixin was originally developed for treatment of chronic obstructive pulmonary disease (COPD), asthma and psoriasis (149). A current phase II clinical trial of navarixin in combination with pembrolizumab is underway in patients with either PD-1 positive refractory NSCLC, castration resistant prostate cancer, or microsatellite stable colorectal cancer (NCT03473925) (150).

##### 3.2.6.2 CXCR1 antagonist reparixin

Reparixin is a small molecule dual antagonist of both CXCR1 and CXCR2 (151, 152). Reparixin was originally evaluated as a drug to prevent graft rejection for pancreatic islet cells (153). *In vitro* studies with reparixin in thyroid cancer found that it also exhibits direct anti-tumor activity (154). In a phase I clinical trial in patients with HER-2 negative metastatic breast cancer, reparixin was well tolerated in combination with paclitaxel chemotherapy (155). However, phase II double blinded clinical trials in triple negative breast cancer patients demonstrated no improvement reparixin in combination with paclitaxel exhibited no additional clinical benefit compared to treatment with paclitaxel alone (NCT02370238) (156).

##### 3.2.6.3 CXCR1/2 antagonist ladarixin

Ladarixin, like reparixin is a dual CXCR11/2 antagonist (157). Preclinical evaluation of ladarixin demonstrated significant activity in a mouse model of pancreatic ductal adenocarcinomaimproved activity compared to either agent alone (148, 158). In an animal model of uveal melanoma administration of ladarixin repolarized TAMs to a M1 phenotype and inhibited tumor cell migration (157). Ladarixin has been used in clinical trials for diabetes, however clinical trials for cancers have not been reported.

##### 3.2.6.4 CXCR2 antagonist AZD5069

AZD5069 is a highly selective small molecule antagonist of CXCR2 receptors that has been shown to inhibit neutrophil migration in patients with COPD (159). It is currently in clinical trials to deplete TANs in the TME in patients with metastatic pancreatic ductal adenocarcinoma and in relapsed metastatic squamous cell carcinoma of the head and neck in combination with ICI (160, 161). In addition, AZD5069 is being evaluated in combination with the androgen receptor antagonist enzalutamide in phase I/II trials in patients with metastatic castration resistant prostate cancer (mCRPC) in the UK (NCT03177187). The combination treatment was well tolerated with no dose limiting toxicities observed. The study observed that 2 out of 15 patients experienced a PR and 10 of 15 patients experienced SD, with responses lasting 2-16 months. Another trial demonstrated that AZD5069 has antitumor activity and depleted TAN density in patients with mCRPC (162).

##### 3.2.6.5 Dual CXCR1/2 antagonist SX-682

SX-682 is another dual CXCR1/2 antagonist, which in rodent models of head and neck cancer have demonstrated suppression of PMN-MDSC accumulation and enhanced tumor infiltration with adoptively transferred NK cells (163, 164). SX-682 is currently being tested in phase I clinical trials in combination with ICI for metastatic melanoma (NCT03161431), and in phase II trials for pancreatic cancer, lung cancer, colon and rectal adenocarcinoma (NCT05604560, NCT05570825, NCT04599140).

### 3.3 CXCR4 blockade to inhibit tumor angiogenesis and metastases

Signaling by the chemokine receptor CXCR4 after binding the chemokine CXCL12 (SCF-1) triggers increased tumor proliferation, survival, and chemotaxis (165). Notably, CXCR4 is overexpressed by many different types of cancers, where it plays a role in tumor metastasis, and also a critical role in mobilizing and recruiting MDSC from bone marrow. Blockade of the CXCR4 signaling is hypothesized to not only decrease tumor angiogenesis but also decrease the number of cancer stem cells and increase mobilization and recruitment of effector T cells into the TME (166) (**Table 7**).

**Table 7:** US clinical trials using CXCR4 targeting drugs for cancer treatment.

CXCR4 blockade	NCT Number	Title	Status	Study Results	Conditions	Interventions	Phases	Enrollment
1	NCT04177810	Plerixafor and Cemiplimab in Metastatic Pancreatic Cancer	Recruiting	No Results Available	Metastatic Pancreatic Cancer	Drug: Cemiplimab|Drug: Plerixafor	Phase 2	21
2	NCT01610999	Pilot Study of Lymphoid Tumor Microenvironmental Dysruption Prior to Autologous Stem Cell Transplantation	Terminated	No Results Available	Chronic Lymphocytic Leukemia|Lymphoma|Multiple Myeloma	Drug: Plerixafor	Phase 1	7
3	NCT03240861	Genetically Engineered PBMC and PBSC Expressing NY-ESO-1 TCR After a Myeloablative Conditioning Regimen to Treat Patients With Advanced Cancer	Recruiting	No Results Available	HLA-A*0201 Positive Cells Present|Locally Advanced Malignant Neoplasm|NY-ESO-1 Positive|Unresectable Malignant Neoplasm|Sarcoma	Other: 18F-FHBG|Biological: Aldesleukin|Drug: Busulfan|Biological: Cellular Therapy|Procedure: Computed Tomography|Biological: Filgrastim|Drug: Fludarabine|Procedure: Leukapheresis|Drug: Plerixafor|Procedure: Positron Emission Tomography	Phase 1	12
4	NCT01977677	Plerixafor After Radiation Therapy and Temozolomide in Treating Patients With Newly Diagnosed High Grade Glioma	Completed	1/3 (33.33%) SAE at Plerixafor 200 mcg/kg/Day	Adult Ependymoblastoma|Adult Giant Cell Glioblastoma|Adult Glioblastoma|Adult Gliosarcoma|Adult Medulloblastoma|Adult Mixed Glioma|Adult Oligodendroglial Tumors|Adult Pineoblastoma|Adult Supratentorial Primitive Neuroectodermal Tumor (PNET)	Radiation: radiation therapy|Drug: temozolomide|Drug: plerixafor|Other: laboratory biomarker analysis|Other: pharmacological study	Phase 1|Phase 2	30
5	NCT00512252	AMD3100 Plus Mitoxantrone, Etoposide and Cytarabine in Acute Myeloid Leukemia	Completed	CR up to 47%, 1 yr Relapse-free Survival 42.9%	Leukemia, Myeloid, Acute	Drug: AMD3100|Drug: Mitoxantrone|Drug: Etoposide|Drug: Cytarabine	Phase 1|Phase 2	52
6	NCT00669669	O6-Benzylguanine-Mediated Tumor Sensitization With Chemoprotected Autologous Stem Cell in Treating Patients With Malignant Gliomas	Terminated	response rate 9.1%, no SAE	Glioblastoma|Gliosarcoma	Radiation: 3-Dimensional Conformal Radiation Therapy|Procedure: Autologous Hematopoietic Stem Cell Transplantation|Drug: Carmustine|Biological: Filgrastim|Procedure: In Vitro-Treated Peripheral Blood Stem Cell Transplantation|Radiation: Intensity-Modulated Radiation Therapy|Other: Laboratory Biomarker Analysis|Drug: O6-Benzylguanine|Drug: Plerixafor|Radiation: Proton Beam Radiation Therapy|Drug: Temozolomide	Phase 1|Phase 2	12
7	NCT01160354	Plerixafor and Clofarabine in Frontline Treatment of Elderly Patients With Acute Myelogenous Leukemia (AML)	Terminated	CR 35.7%, PR 7.1% (Plerixafor 400 mcg/kg + Clofarabine),	Acute Myelogenous Leukemia	Drug: Plerixafor|Drug: Clofarabine	Phase 1|Phase 2	22
8	NCT01352650	Decitabine and Plerixafor in Elderly Acute Myeloid Leukemia (AML)	Completed	No Results Available	Acute Myeloid Leukemia	Drug: plerixafor|Drug: decitabine	Phase 1	71
9	NCT01027923	IV Plerixafor With Mitoxantrone Etoposide and Cytarabine for Acute Myeloid Leukemia (AML)	Terminated	No Results Available	Leukemia, Myeloid, Acute	Drug: Plerixafor|Drug: Mitoxantrone|Drug: Etoposide|Drug: Cytarabine	Phase 1	6
10	NCT00943943	Granulocyte-colony Stimulating Factor (G-CSF) and Plerixafor Plus Sorafenib for Acute Myelogenous Leukemia (AML) With FLT3 Mutations	Completed	No Results Available	Acute Myelogenous Leukemia|Leukemia	Drug: G-CSF|Drug: Plerixafor|Drug: Sorafenib	Phase 1	33
11	NCT05088356	Reduced Intensity Allogeneic HCT in Advanced Hematologic Malignancies w/T-Cell Depleted Graft	Recruiting	No Results Available	Allogeneic Hematopoietic Cell Transplantation (HCT)|Advanced Hematologic Malignancies|Acute Leukemia|Chronic Myelogenous Leukemia|Myelodysplastic Syndromes|Myeloproliferative Disorders	Drug: Purified regulatory T-cells (Treg) plus CD34+ HSPC|Drug: Fludarabine|Drug: Melphalan|Device: CliniMACS CD34 Reagent System|Drug: Tacrolimus|Drug: Cyclophosphamide|Drug: Plerixafor|Drug: Filgrastim granulocyte colony-stimulating factor (G-CSF) or equivalent	Phase 1	24
12	NCT00906945	Chemosensitization With Plerixafor Plus G-CSF in Acute Myeloid Leukemia	Completed	45 day CR 30%, Relapse Free-survival Rate 75% at 2 yrs. SAE 2/3 (66.67%) at dose level 2	Leukemia, Myeloid, Acute	Drug: G-CSF|Drug: Plerixafor|Drug: Mitoxantrone|Drug: Etoposide|Drug: Cytarabine	Phase 1|Phase 2	39
13	NCT00903968	Combination Plerixafor (AMD3100)and Bortezomib in Relapsed or Relapsed/Refractory Multiple Myeloma	Completed	SD up to 100% in dose level 1 and 5. Time to Progression (TTP) 12.6 mo, Duration of Response phase 2 (DOR) 12.9 mo	Multiple Myeloma	Drug: Plerixafor|Drug: bortezomib|Drug: Dexamethasone	Phase 1|Phase 2	58
14	NCT01696461	A Phase II Study Evaluating the Safety and Efficacy of Subcutaneous Plerixafor	Completed	No Results Available	Related Donors Donating PBSC to a Family Member|Acute Myelogenous Leukemia|Acute Lymphoblastic Leukemia|Myelodysplastic Syndrome|Chronic Myelogenous Leukemia|Non-Hodgkin's Lymphoma|Hodgkin's Disease|Chronic Lymphocytic Leukemia	Drug: Plerixafor	Phase 2	128
15	NCT00990054	Study of Plerixafor Combined With Cytarabine and Daunorubicin in Patients With Newly Diagnosed Acute Myeloid Leukemia	Completed	No Results Available	Acute Myeloid Leukemia	Drug: Plerixafor	Phase 1	36
16	NCT03746080	Whole Brain Radiation Therapy With Standard Temozolomide Chemo-Radiotherapy and Plerixafor in Treating Patients With Glioblastoma	Recruiting	No Results Available	Glioblastoma|Glioblastoma With Primitive Neuronal Component|Gliosarcoma|Malignant Glioma|Oligodendroglial Component Present	Drug: Plerixafor|Drug: Temozolomide|Radiation: Whole-Brain Radiotherapy (WBRT)|Radiation: Radiation Therapy	Phase 2	20
17	NCT01339039	Plerixafor (AMD3100) and Bevacizumab for Recurrent High-Grade Glioma	Terminated	No Results Available	High Grade Glioma: Glioblastoma (GBM)|High Grade Glioma: Gliosarcoma|Anaplastic Astrocytoma (AA)|Anaplastic Oligodendroglioma (AO)|Mixed Anaplastic Oligoastrocytoma (AOA)	Drug: Plerixafor|Drug: Bevacizumab|Procedure: Surgery	Phase 1	26
18	NCT01373229	Lenalidomide + Plerixafor in Previously Treated Chronic Lymphocytic Leukemia (CLL)	Completed	PFS 11 mo, OS 5.5 mo, SAE 93.33%	Leukemia, Lymphocytic, Chronic, B-Cell	Drug: Lenalidomide + Plerixafor (+ Rituximab)	Phase 1	21
19	NCT01065129	Plerixafor and Granulocyte Colony-stimulating Factor (G-CSF) in Combination With Azacitidine for the Treatment of Myelodysplastic Syndrome (MDS)	Completed	No Results Available	Myelodysplastic Syndromes	Drug: G-CSF|Drug: Plerixafor|Drug: Azacitidine	Phase 1	28
20	NCT00694590	Study of AMD3100 (Plerixafor) and Rituximab in Patients With Chronic Lymphocytic Leukemia or Small Lymphocytic Lymphoma	Completed	No Results Available	Chronic Lymphocytic Leukemia (CLL)|Small Lymphocytic Lymphoma (SLL)	Drug: plerixafor	Phase 1	24
21	NCT01319864	POETIC Plerixafor as a Chemosensitizing Agent for Relapsed Acute Leukemia and MDS in Pediatric Patients	Completed	No Results Available	Relapsed/Refractory AML|Relapsed/Refractory ALL|Secondary AML/MDS|Acute Leukemia of Ambiguous Lineage|AML|ALL	Drug: Plerixafor Dose Escalation	Phase 1	20

#### 3.3.1 AMD3100

AMD3100 (plerixafor) is currently the only FDA approved CXCR4 inhibitor. This drug was initially developed for treatment and prevention of HIV, but has now also been approved for treatment of non-Hodgkin lymphoma (NHL) and multiple myeloma (MM) (167, 168). Use of AMD3100 in combination with the anti-VEGFR2 antibody ramucirumab in a mouse model of colorectal cancer significantly reduced recruitment of immune suppressive monocytes, as the study demonstrated that depletion of immune suppressive Ly6C^low^ monocytes by CXCR4 blockade was associated with enhanced treatment efficacy of ramucirumab (169, 170). Administration of AMD3100 was also associated with increased CD8+ T cell infiltration and synergistic activity when combined with ICI (171) Use of AMD3100 in NHL and MM suppressed tumor growth and metastasis and was associated with converting Tregs to a Th1 phenotype and enhancing CD8+ T cell infiltration (172).

Another mechanism of AMD3100 antitumor activity was to block CXCR4+ tumor cells from interacting with CXCL12 produced by cancer associated fibroblasts (173). Use of AMD3100 in combination with ICI in patients with microsatellite unstable pancreatic or colorectal cancer demonstrated enhanced B cell and T cell antitumor responses (174). Clinical trials evaluating AMD3100 include phase II trials for metastatic pancreatic cancer, phase I and II trials for glioma, and phase I and II trials for hematopoietic malignancies. The proposed mechanism targeted in these trials is to sensitize the TME to chemotherapy by blocking the CXCR4 and CXCR/2 axes (175). Other applications of AMD3100 is as a hematopoietic stem cell (HSC) mobilizing agent (typically in combination with G-CSF) for hematopoietic stem cell transplantation (176).

#### 3.3.2 BPRCX807

BPRCX807 is a selective CXCR4 antagonist that has shown activity in mouse models of hepatocellular carcinoma (177). In these models BPRCX807 prevented tumor cell migration and limited the development of metastases. Another activity of BPRCX807 is to reprogram immune suppressive TAMs to a more an immunostimulatory M1 phenotype, while at the same time promoting CD8+ T cell infiltration into tumors (177). Early mouse studies provide support for further investigation of CXCR4 blockade as a combination agent along with ICI (172).

## 4 Metabolic reprogramming to target myeloid suppressor cells

### 4.1 IDO inhibitors

The enzyme indoleamine 2,3-dioxygenase 1 (IDO1) converts the essential amino acid tryptophan (Trp) to kynurenine (Kyn), thereby leading to an overall depletion of this critical amino acid within the TME and tumor draining lymph nodes (178). Overexpression of IDO1 is considered an important driver of tumor associated immune suppression and a key to establishing immune tolerance of cancer antigens (179, 180). High intratumoral IDO1 expression is correlated with poor prognosis in melanoma, ovarian cancer, colorectal cancer, and lung cancers (181, 182). In ovarian cancer, high IDO1 expression also correlates with increased drug resistance (183). A high ratio of tryptophan to kynurenine in blood is also associated with a poorer prognosis in some cancers (184–187).

High levels of IDO1 expression by cancer cells can also drive MDSC expansion (188); Moreover, MDSCs also overexpress IDO1, triggering a positive feedback loop that reinforces and sustains the immune suppressive TME (189). Local depletion of tryptophan by IDO leads to cell cycle arrest and apoptosis of effector T cells in tumor tissues (190). In addition, IDO1 positive MDSCs also contribute to T cell exhaustion through IL-6 secretion. The local buildup of kynurenine concentrations within the TME also triggers deleterious alterations in the metabolic properties of tumor infiltrating T cells and converts CD4 effector cells to Tregs (190–193). Evaluation of IDO inhibitors in preclinical models demonstrated a reduction of IDO1+ MDSCs within the TME and measurable reduction of Kyn concentrations (178). Taken together, these properties make IDO1 a promising target for reversing immune suppression through metabolic reprogramming of the TME.

#### 4.1.1 Epacadostat as an IDO synthesis inhibitor

Van den Eynde et al. summarized the many clinical trials evaluating epacadostat up to 2020 and in their paper discussed why the outcomes of these trials have been largely negative. The majority of these trials have evaluated epacadostat in combination with checkpoint blockade (CTLA4, PD-L1 or PD-1) and have to date failed to demonstrate any meaningful clinical benefit. It was concluded therefore that epacadostat did not improve ICI, as confirmed in at least 12 clinical trials (194, 195). Due to these poor results, remaining clinical trials with epacadostat have been withdrawn, downsized or suspended.

#### 4.1.2 Navoximod (GDC-0919)

Navoximod has been evaluated clinically as a monotherapy or in combination with atezolizumab (NCT02048709, NCT02471846, NCT05469490, and these trials demonstrated that the navoximod was well tolerated and decreased plasma Kyn concentrations in a dose dependent manner (181). However, there was no clear tumor response benefit in the navoximod combination therapy arm when compared to treatment with atezolizumab alone (196).

### 4.2 Repurposed beta blockers as MDSC depleting agents

In addition to stimulation of cortisol release, chronic stress from inflammation in cancer is associated with prolonged activation of the sympathetic nervous system (53). Chronic adrenergic activation and release of catecholamines, primarily norepinephrine (Nor), has been associated with MDSC mobilization from the bone marrow and acquisition of greater immune suppressive properties, leading to both systemic and local immune suppression (197, 198). A consequence of increased Nor concentrations is higher concentrations of both MDSCs and TAMs in tumor tissues. For example, activation of β2-adrenergic receptor (β-AR) signaling was shown to upregulate STAT3 and NFk-b signaling pathways which drive development of immune suppressive MDSC and TAMs (199). Activation of β-AR signaling has been shown to polarize macrophages to an immunosuppressive M2 phenotype in a rodent breast cancer model (200, 201). Adrenergic signaling in tumor cells themselves can also be triggered by tumor hypoxia (202).

#### 4.2.1 Propranolol as non-selective β-blocker

Use of non-selective beta blockers such as propranolol have been investigated for their ability to reprogram immune suppressive cells within the TME (200, 203). Blocking β-AR signaling by MDSCs with propranolol can prevent their mobilization from the bone marrow (204). In addition, propranolol treatment reprograms MDSCs to a less immune suppressive state by blocking STAT3 signaling (205). This effect has been demonstrated in rodent models, where treatment with propranolol reduces MDSC mobilization and accumulation within the TME, accompanied by inhibition of tumor growth and metastasis (206). In rodent models, treatment with propranolol blocked the accumulation of M2 macrophages in metastatic breast cancer and inhibited metastases (53). In a spontaneous melanoma mouse model, propranolol treatment significantly reduced intratumoral accumulation of neutrophils, immune suppressive inflammatory (CD11c-Ly6C^hi^Ly6G-) macrophages and DCs in both the primary tumor and metastatic lesions (207). Multiple rodent studies and recent clinical trials in dogs and human patients have demonstrated the ability of propranolol 3008 treatment to improve responses to radiation therapy for glioma, 3009 breast cancer, and pancreatic cancer (200, 208) (**Table 8**).

**Table 8:** US clinical trials using propranolol as cancer treatment.

β-AR blockade	NCT Number	Title	Status	Study Results	Conditions	Interventions	Phases	Enrollment
1	NCT01847001	Study of Propranolol in Newly Diagnosed Breast Cancer Patients Undergoing Neoadjuvant Chemotherapy	Completed	Propranolol + Neoadjuvant Chemotherapy SAE 10%	Locally Advanced Malignant Neoplasm|Breast Cancer	Drug: Propranolol|Other: DOT imaging|Drug: Paclitaxel|Drug: Nab-paclitaxel|Drug: Trastuzumab|Drug: Pertuzumab|Drug: Doxorubicin|Drug: Cyclophosphamide|Procedure: Surgery|Drug: Premedication|Drug: Anti-nausea therapy|Drug: Pegfilgrastim	Phase 2	10
2	NCT01308944	Therapeutic Targeting of Stress Factors in Ovarian Cancer Patients	Completed	No Results Available	Invasive Epithelial Ovarian Cancer|Primary Peritoneal Carcinoma|Fallopian Tube Cancer	Drug: Propranolol	Phase 1	24
3	NCT02165683	Use of Propranolol to Reduce FDG Uptake in Brown Adipose Tissue in Pediatric Cancer Patients PET Scans	Completed	No Results Available	Pediatric Cancer	Drug: Propranolol	Phase 1	10
4	NCT01902966	Feasibility - Beta Adrenergic Blockade (BB) in Cervical Cancer (CX)	Terminated	dose escalation 40 mg by mouth twice a day, SAE 20%	Cervical Cancer	Drug: Propranolol|Behavioral: Diary|Behavioral: Relaxation Audio Recording|Behavioral: Questionnaires	Not Applicable	6
5	NCT04848519	Propranolol Hydrochloride and Pembrolizumab for the Treatment of Recurrent or Metastatic Urothelial Cancer	Recruiting	No Results Available	Recurrent or Metastatic Urothelial Cancer	Drug: Pembrolizumab|Drug: Propranolol Hydrochloride	Phase 2	25
6	NCT03152786	Propranolol Hydrochloride in Treating Patients With Prostate Cancer Undergoing Surgery	Suspended	No Results Available	Prostate Carcinoma	Other: Laboratory Biomarker Analysis|Drug: Propranolol Hydrochloride|Other: Questionnaire Administration|Other: Survey Administration	Phase 2	50
7	NCT05651594	Propranolol in Combination With Pembrolizumab and Standard Chemotherapy for the Treatment of Unresectable Locally Advanced or Metastatic Esophageal or Gastroesophageal Junction Adenocarcinoma	Recruiting	No Results Available	Unresectable Locally Advanced or Metastatic Esophageal or Gastroesophageal Junction Adenocarcinoma	Procedure: Biopsy|Procedure: Biospecimen Collection|Procedure: Computed Tomography|Drug: Fluorouracil|Drug: Leucovorin|Drug: Oxaliplatin|Biological: Pembrolizumab|Drug: Propranolol Hydrochloride|Other: Questionnaire Administration	Phase 2	40
8	NCT01504126	Propranolol Hydrochloride and Chemotherapy in Treating Patients With Ovarian, Primary Peritoneal, or Fallopian Tube Cancer	Completed	No Results Available	Ovarian, Primary Peritoneal, or Fallopian Tube Cancer	Drug: Chemotherapy|Drug: Propranolol Hydrochloride|Other: Quality-of-Life Assessment|Procedure: Therapeutic Conventional Surgery	Early Phase 1	32
9	NCT04682158	Propranolol With Standard Chemoradiation for Esophageal Adenocarcinoma	Recruiting	No Results Available	Esophageal Adenocarcinoma	Drug: Carboplatin|Radiation: 3 Dimensional Conformal Radiation Therapy|Drug: Propranolol|Radiation: Intensity Modulated Radiation Therapy|Drug: Paclitaxel	Phase 2	60
10	NCT03384836	Propranolol Hydrochloride and Pembrolizumab in Treating Patients With Stage IIIC-IV Melanoma That Cannot Be Removed by Surgery	Recruiting	No Results Available	Stage IIIC Cutaneous Melanoma AJCC v7|Stage IV Cutaneous Melanoma AJCC v6 and v7	Other: Laboratory Biomarker Analysis|Biological: Pembrolizumab|Drug: Propranolol Hydrochloride	Phase 1|Phase 2	47
11	NCT00967226	Propranolol Versus Prednisolone for Treatment of Symptomatic Hemangiomas	Terminated	Propranolol SAE 1/11 (9.09%)	Hemangioma of Infancy	Drug: propranolol|Drug: Prednisolone	Phase 2	19
12	NCT05312255	Non-chemotherapeutic Interventions for the Improvement of Quality of Life and Immune Function in Patients With Multiple Myeloma	Recruiting	No Results Available	Plasma Cell Myeloma|Recurrent Plasma Cell Myeloma|Refractory Plasma Cell Myeloma|Smoldering Plasma Cell Myeloma	Behavioral: Behavioral Intervention|Drug: Beta-Adrenergic Antagonist|Drug: Propranolol|Other: Quality-of-Life Assessment|Other: Questionnaire Administration|Other: Resistance Training|Other: Short-Term Fasting	Not Applicable	150
13	NCT01074437	Corticosteroids With Placebo Versus Corticosteroids With Propranolol Treatment of Infantile Hemangiomas (IH)	Terminated	Has Results	Hemangioma	Drug: Prednisolone (Corticosteroid)|Drug: Propranolol|Drug: Placebo	Phase 2	9
14	NCT05479123	Assessing the Impact of Dosage Frequency of Propranolol on Sleep Patterns in Patients With Infantile Hemangiomas	Recruiting	No Results Available	Infantile Hemangioma	Drug: Propranolol three times a day|Drug: Propranolol twice a day|Drug: Timolol	Phase 4	174
15	NCT01056341	Study to Demonstrate the Efficacy and Safety of Propranolol Oral Solution in Infants With Proliferating Infantile Hemangiomas Requiring Systemic Therapy	Completed	Propranolol 3mg/kg/d 6 Months, 60.4% resolution. SAE 4.95% for Propranolol 3mg/kg/day for 6 months	Infantile Hemangioma	Drug: Propranolol|Drug: Placebo	Phase 2|Phase 3	512
16	NCT01265576	Study of Sorafenib With or Without VT-122 in Patients With Hepatocellular Carcinoma (HCC)	Unknown status	No Results Available	HCC	Drug: Sorafenib|Drug: VT-122 (propranolol plus etodolac)|Drug: Placebo	Phase 2	20

In a phase II trial in patients with metastatic breast cancer it was found that in tumor tissues from propranolol treated patients there was upregulated expression of genes associated with classical dendritic cells and an increase in M1 macrophage polarization, along with an increase in CD69+ activated TAMs (209). Phase I trials of propranolol with pembrolizumab in patients with metastatic and locally advanced melanoma showed encouraging responses and the combination therapy to be well tolerated (210). In the USA there are currently 17 trials investigating propranolol in breast cancer, cervical cancer, prostate, esophageal, infantile hemangioma and hepatocellular carcinoma. Further studies are warranted to elucidate the clinical benefit of propranolol as a repurposed immunotherapy for TME reprogramming (**Table 8**).

### 4.3 Tyrosine kinase inhibitors

Tyrosine kinase inhibitors (TKIs), especially early generation non-specific TKIs such as sunitinib, have been shown to alter the immune suppressive TME, in part by reprogramming TAMs from M2 to M1 phenotypes, by reducing total TAM infiltrates and by blocking the accumulation of MDSCs and TANs (211–213). Tyrosine kinase receptors are extremely diverse family of receptors and there are >40 FDA approved TKI drugs. These TKIs are categorized according to the main receptor targeting sites which include, anaplastic lymphoma kinase (ALK), epidermal growth factor receptor (EGFR), FMS-like tyrosine kinase 3 (FLT3), vascular endothelial growth factor (VEGFR), and tropomyosin receptor kinase (TRK) (214). The positive clinical benefits observed when TKIs are combined with ICI indicate that the TKI impact on the TME is substantial and complementary to ICI therapy. The list of multi-target TKIs is quite extensive, therefore few are selected here for discussion to illustrate their potential as immunotherapy drugs.

#### 4.3.1 VEGFR targeted TKIs

##### 4.3.1.1 Sunitinib (*SU011248*, Sutent*)*

Sunitinib is a small molecule inhibitor that targets multiple kinases, with inhibitory effects against signaling by VEGFR, PDGFR and c-kit (215). It is an FDA approved agent for treatment of renal cell carcinoma and gastrointestinal stromal tumors (216). To date there are currently 270 US trials of sunitinib to treat, alone or in combination, many different cancers, including breast, hepatic, lung, and renal cancers. Early generation, multi-function TKIs such as sunitinib have been shown to exert impressive immune modulatory effects (217, 218). For example, sunitinib has been shown to deplete both MDSC and Tregs, in part by inhibiting STAT3 signaling (219); and in clinical trials positive responses to treatment with sunitinib has been associated with Treg depletion (220, 221).

##### 4.3.1.2 Sorafenib (Nexavar, BAY 43-9006)

Sorafenib is another small molecule multi-kinase inhibitor, which in hepatocellular carcinoma has shown clinical benefit and antitumoral activity that is associated with immune remodeling of the TME (222). For example, treatment with sorafenib has been reported to selectively decrease Tregs numbers without impacting effector T cell numbers (223). Sorafenib has been shown to regulate the differentiation DCs in the TME (224) and to repolarize M2 TAMs to an M1 phenotype through inhibition of miR-101 expression and reduction of TGF-β secretion. Sorafenib has also been reported to induce secretion of pro-inflammatory cytokines such as IL-12 by TAMs (225, 226) and to decrease expression of PD-L1 on MDCS and plasmacytoid DCs (227, 228). There are 430 clinical trials registered in the US using sorafenib in cancer patients ranging from phase I to phase IV clinical trials, with many focused on renal cell carcinoma.

##### 4.3.1.3 Lenvatinib (E7080, Lenvima)

Lenvatinib is another multitarget TKI that has shown in phase III trials clinical benefit as reflected by significantly increased overall survival times in patients with hepatocellular carcinoma (227). There are currently 128 registered lenvatinib clinical trials in the USA, with multiple phase I through phase III trials for treatment of thyroid cancer, renal cell carcinoma, hepatocellular carcinoma and melanoma. The antitumor activity of lenvatinib is also heavily linked to its anti-angiogenic properties (229). In addition, Lenvatinib has been shown to reduce TAMs and increase IFNγ secreting CD8 effector T cells in tumor tissues in a mouse model of colon carcinoma (230).

#### 4.3.2 EGFR targeted TKI

EGFR targeted TKIs disrupt the immune suppressive TME by several mechanism including blocking cancer cell migration and nutrient delivery through targeting of endothelial cells and suppressing pericyte coverage (231). Highly proliferative cancer stem cells also express EGFR and can be inhibited by EGFR targeted TKIs (232). For example, EGFR-mutated NSCLC is known to be especially sensitive to treatment with EGFR TKIs (233); and these TKIS are therefore often a first line treatment for this cancer (233, 234). Many EGFR TKI drugs have been developed, and first-generation drugs such as gefitinib, erlotinib, and afatinib are approved for the treatment of EGFR mutated NSCLC. Currently third generation EGFR TKI drugs are being investigated as monotherapy and in combination with chemotherapy (213). There are currently around 8 FDA approved EGFR targeted TKI (235) with over 1200 total clinical trials in the US ranging from phase I to phase IV.

## 5 Future opportunities for myeloid cells as targets in cancer immunotherapy

Many different strategies targeting immune suppressor cells within the TME to reverse or ameliorate immune suppression have been evaluated. To date, the most successful strategies have been those targeting MDSCs, including the use of multi-function TKIs and repurposed beta blockers. For reprogramming TAMs, the most studied targets to date have been CSF-R1 inhibitors, either as biologics or targeted agents, though clinical responses to date have not been impressive (201, 236, 237). Other strategies have been even less successful, including the use of arginase and IDO inhibitors to reprogram metabolic pathways used by TAMs and tumor cells (194, 238). In the future, the most successful rational strategies will likely employ drugs and biologics targeting multiple different complementary pathways of tumor immune evasion, to block non-redundant mechanisms and pathways. Such combination strategies may also include creative uses of radiation therapy to enhance tumor immunogenicity, while MDSC or inflammatory monocyte targeted drugs can be used to relieve radiation induced inflammatory responses. Other gains will undoubtedly be realized when newer drugs and biologics with greater activity or more specific targeting of myeloid cell pathways enter the clinic. Thus, it is likely that we will see greater use of myeloid targeted agents as part of a more comprehensive strategy and platform for cancer immunotherapy (235).

## Author contributions

JC, LC, SD collection and assembly of data, conception design and manuscript writing and revision. JC and LC are equal contribution first authors. All authors contributed to the article and approved the submitted version. 
